# Synthesis, structure, photochemical and electrochemical properties of α-germyl ferrocenyl ketones

**DOI:** 10.1039/d5dt02029h

**Published:** 2025-11-06

**Authors:** Madeleine Heurix, Petr Harmach, Ana Torvisco, Roland C. Fischer, Mathias Wiech, Ivana Císařová, Georg Gescheidt, Petr Štěpnička, Michael Haas

**Affiliations:** a Institute of Inorganic Chemistry, Graz University of Technology Stremayrgasse 9/IV 8010 Graz Austria michael.haas@tugraz.at; b Department of Inorganic Chemistry, Faculty of Science, Charles University Hlavova 2030 128 00 Prague Czech Republic stepnic@natur.cuni.cz; c Institute of Physical and Theoretical Chemistry, Graz University of Technology Stremayrgasse 9/I 8010 Graz Austria

## Abstract

Owing to their well-defined and tunable properties, acylgermanes are attractive photoinitiators for polymerization reactions. This paper focuses on the previously unexplored acylgermanes containing the ferrocenylcarbonyl group. For example, compound FcC(O)Ge(SiMe_3_)_3_ (4, Fc = ferrocenyl) was synthesized from the reaction of (chlorocarbonyl)ferrocene with the germanide K[Ge(SiMe_3_)_3_] generated from KO*t*Bu and tetrakis(trimethylsilyl)germane (1) in a continuous flow setup. In contrast, a complete replacement of the silyl groups in 1 in the presence of KO*t*Bu and KF under conventional conditions produced the tetraacylgermane (FcC(O))_4_Ge (5). Additionally, mixed-acyl compounds, (FcC(O))_*n*_Ge(C(O)Mes)_4−*n*_ (*n* = 1: 6, *n* = 2: 7; Mes = mesityl), were obtained by salt metathesis of the respective germenolates K_*n*_Ge(C(O)Mes)_4−*n*_ (2 and 3) with FcC(O)Cl. All compounds, along with the model silane FcC(O)Si(SiMe_3_)_3_ (4Si), prepared from *in situ*-generated KSi(SiMe_3_)_3_ and FcC(O)Cl, were fully characterized by elemental analysis, NMR and UV-vis spectroscopy, single-crystal X-ray diffraction analysis, and their electrochemical properties were studied using voltammetric techniques. Compounds bearing exclusively ferrocenoyl substituents were photoinert, whereas mixed ferrocenoyl/mesitoyl derivatives showed α-cleavage upon visible-light irradiation. Remarkably, this reactivity extended up to 550 nm, representing the longest wavelength reported for α-cleavage in acylgermanes and underscoring their potential for mild, visible-light-driven applications. Preliminary experiments suggested a limited reactivity of 4 and 4Si, likely due to steric effects. Only the reaction of the silane with benzil at elevated temperature produced siladioxacyclopentene FcC(SiMe_3_)_2_{Si(OSiMe_3_)O_2_C_2_Ph_2_} (8).

## Abstract 2

We report the first synthesis of α-germyl ferrocenyl ketones, revealing photoinert all-ferrocenoyl derivatives and visible-light-induced α-cleavage (up to 550 nm) for mixed acyl species, alongside distinct multiferrocenyl redox behavior.

## Graphical abstract

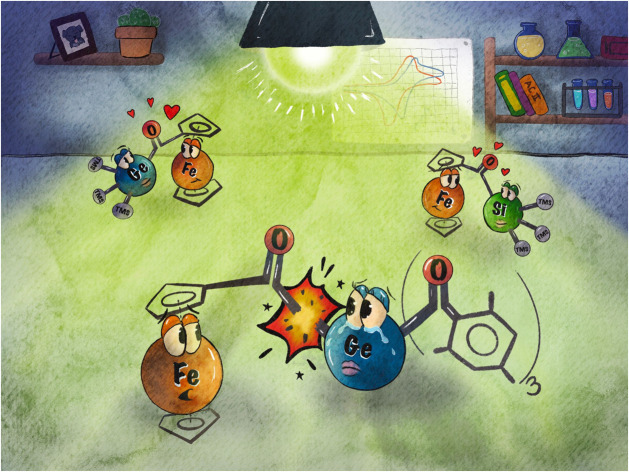

## Introduction

Photoinitiators (PIs) are the key components in light-driven polymerization processes, enabling high spatial and temporal control over radical generation in applications ranging from the production of coatings and adhesives to advanced biomedical materials and 3D printing.^1^ Over the past decade, acylgermanes have emerged as a powerful class of visible-light photoinitiators due to their favorable absorption profiles, low toxicity, and efficient α-cleavage (Norrish type I) behavior under mild conditions.^2,3–6^ Their utility has been demonstrated in both radical^7^ and hybrid polymerization systems,^8^ offering an attractive alternative to traditional photoinitiators based on benzoin ethers, α-hydroxyketones, or acylphosphine oxides.^9^

However, despite significant progress, acylgermanes suffer from key limitations. Many require bulky aryl groups to suppress premature oxidation and improve radical stability, which leads to poor solubility and limited tunability of their electronic properties. The incorporation of additional redox-active or photoresponsive groups could enhance or modulate their behavior in photochemical or redox transformations.

In this context, we wanted to explore ferrocenyl-substituted germyl ketones that combine a photo-^10^ and redox-active ferrocene unit with a germyl group at the α-position. The motivation for this design is fourfold: (i) the ferrocenyl group provides a well-defined and reversible redox handle that can participate in electron transfer events;^11^ (ii) the germyl group can stabilize radical intermediates and modulate the electronic environment of the ketone; (iii) the combined organometallic framework offers access to dual-function materials with potential as tunable photoinitiators, redox sensors, or photocatalytic scaffolds;^12^ and (iv) to the best of our knowledge, only two publications report on the synthesis of simple α-germyl ferrocenyl ketones FcC(O)GeR_3_ (R = Me, Ph; Fc = ferrocenyl) and their electrochemical behavior.^13,14^

## Results and discussion

For this work, tetrakis(trimethylsilyl)germane (1), potassium tris(2,4,6-trimethylbenzoyl)germenolate (2), and dipotassium bis(2,4,6-trimethylbenzoyl)bisgermenolate (3) were selected as the starting materials for further derivatizations. These compounds offer a promising platform for the introduction of one to four ferrocenoyl groups to the germanium center.^5,6,15^

### Tetrakis(trimethylsilyl)germane (1) as the starting material

**Scheme 1:**
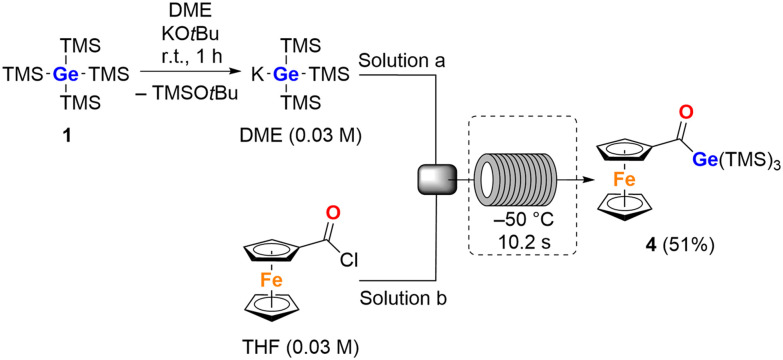
Flow reaction route toward monoferrocenoyl derivative 4 (DME = 1,2-dimethoxyethane).

Our initial objective was to explore the reactivity of 1. To this end, germane 1 was dissolved in 1,2-dimethoxyethane (DME), and solid KO*t*Bu was added. The reaction mixture immediately turned reddish, indicating the onset of the reaction. The resulting solution, containing K[Ge(SiMe_3_)_3_],^16^ was transferred to an equimolar amount of (chlorocarbonyl)ferrocene. The reaction was performed in various solvents, ranging from polar (THF, DME, and Et_2_O) to nonpolar (toluene or *n*-pentane), and at temperatures between −70 °C and 0 °C. However, under all tested conditions, we observed the formation of at least two different acylgermanes along with the backformation of the starting material 1, suggesting that multiple silyl group abstractions were taking place. In order to circumvent these limitations and achieve more reliable control over stoichiometry and reaction kinetics, we made use of a flow chemistry approach. The use of a continuous flow setup enabled precise regulation of reagent mixing, residence time, and temperature, which significantly improved the reproducibility and selectivity of the transformation (for details, see SI).^17^ To our delight, we achieved a significantly more selective reaction toward the targeted monoferrocenyl derivative 4. After aqueous work-up and recrystallization from acetone at −30 °C, compound 4 was isolated in a good yield (Scheme 1).

Compound 4 was characterized using ^1^H, ^13^C, and ^29^Si NMR spectroscopy in C_6_D_6_ as the solvent. The most notable feature in the ^13^C NMR spectrum of 4 is the considerably downfield-shifted signal due to the carbonyl C-atom at *δ*_C_ 232.1, which is characteristic of carbonyl groups directly bonded to a germanium atom^18^ (see *δ*_C_ 178.3 for FcC(O)Cl in CDCl_3_).^19^ All other analytical data are consistent with the proposed structure (see Experimental section; copies of the NMR spectra are provided in the SI).

**Fig. 1:**
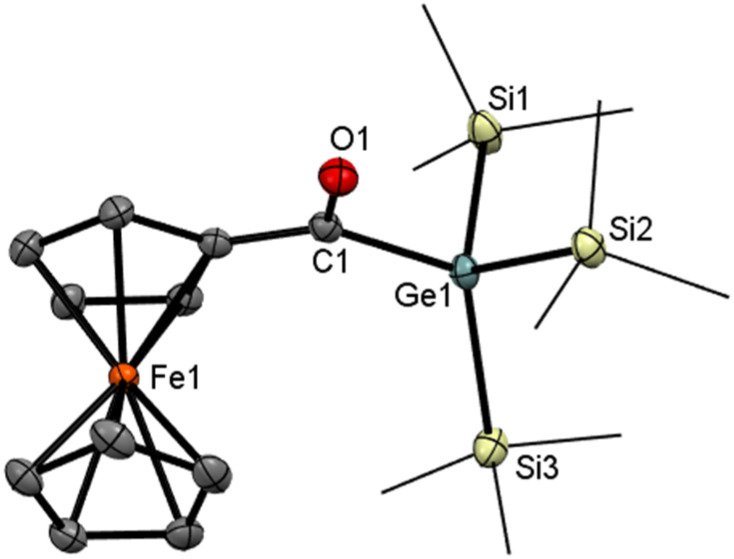
ORTEP representation of compound 4. Thermal ellipsoids are depicted at the 50% probability level. Hydrogen atoms are omitted, and methyl groups are wireframed for clarity. Selected bond lengths (Å) and bond angles (°) with estimated standard deviations: Ge(1)–C(1) 2.3913(3), Ge(1)–Si(1) 2.3861(4), Ge(1)–Si(2) 2.3913(3), Ge(1)–Si(3) 2.3877(4), C(1)–O(1) 1.2256(12), C(1)–C(2) 1.4703(16).

Upon cooling a concentrated acetone solution of 4 to −30 °C, crystals suitable for single-crystal X-ray analysis were formed. The molecular structure is depicted in Fig. 1. Compound 4 crystallizes with the symmetry of the tetragonal space group *I*4_1_/*a* and one molecule per the asymmetric unit. The ferrocene unit in the structure of 4 has its regular geometry with similar Fe–C distances and parallel cyclopentadienyl rings, which assume an eclipsed conformation (Fe–C 2.0362(12)–2.0549(12) Å, tilt angle: 3.43(7)°). The acyl moiety, {C1,O1,Ge1}, is coplanar with its bonding cyclopentadienyl ring (interplanar angle: 0.91(10)°), and the C=O bond length (1.2256(12) Å) is consistent with the data reported for FcC(O)GeR_3_ (R = Me, Ph; 1.223(10) Å).^13^

**Scheme 2:**
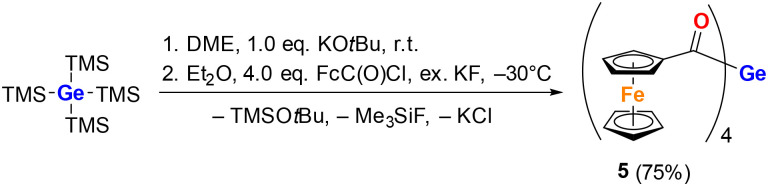
Synthesis of tetraferrocenoyl derivative 5 (Fc = ferrocenyl).

Unselective batch reactions suggesting multiple silyl group abstractions (*vide supra*) convinced us that the tetraacylgermane synthesis with (chlorocarbonyl)ferrocene might indeed be possible. In order to push the system to a complete conversion, we added an excess of KF to *in situ* generate Me_3_SiF, which is gaseous. Consequently, compound 1 was dissolved in DME, and solid KO*t*Bu was added. Subsequently, this solution was added to a Et_2_O solution containing a fourfold molar amount of (chlorocarbonyl)ferrocene and an excess of dry KF (for good reproducibility, at least ten molar equivalents of KF relative to 1 had to be used) at −30 °C. As expected, the tetraferrocenyl derivative 5 was formed in excellent yields and was isolated *via* simple recrystallization from acetone (Scheme 2).

**Fig. 2:**
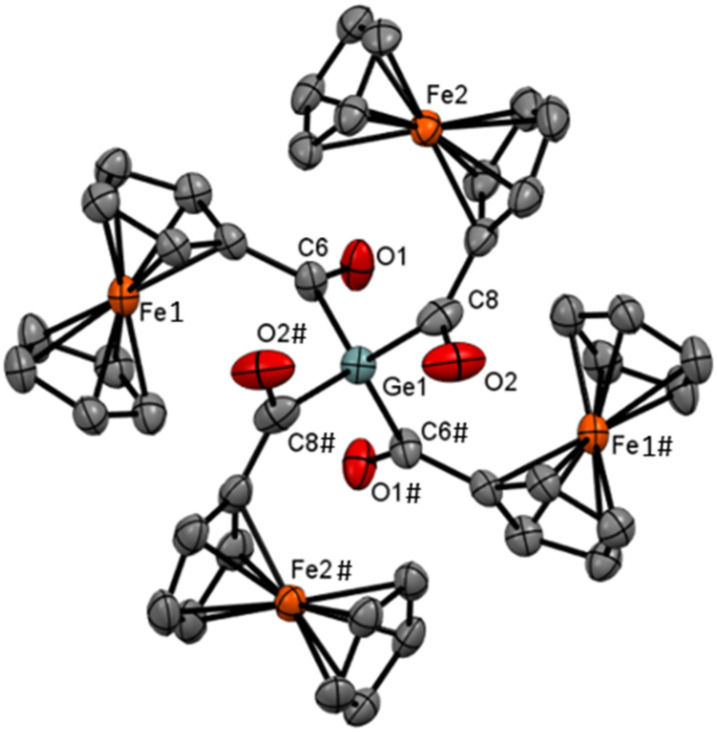
ORTEP representation of compound 5. Thermal ellipsoids are depicted at the 50% probability level. Hydrogen atoms are omitted for clarity. Selected bond lengths (Å) and bond angles (°) with estimated standard deviations: Ge(1)–C(6#) 2.025(4), Ge(1)–C(8#) 2.018(5), O(1#)–C(6#) 1.221(5), O(2#)–C(8#) 1.232(7), C(6#)–C(16#) 1.454(6), C(8#)–C(14#) 1.450(8).

Compound 5 was characterized by ^1^H and ^13^C NMR spectroscopy. The ^13^C NMR spectrum displays an even more pronounced downfield shift for the carbonyl carbon to *δ*_C_ 222.3. Single crystals used for X-ray diffraction analysis were obtained by slow evaporation of a diluted acetone solution at room temperature. The molecular structure is depicted in Fig. 2. Compound 5 crystallizes in the monoclinic space group *C*2/*c* with the molecule residing over the crystallographic two-fold axis, so that only half of the molecule is structurally independent. The Ge–C distances are quite similar (2.025(5) and 2.017(5) Å), but the C–Ge–C angles vary between 99° and 115°, reflecting some steric crowding. The C=O distances (≈1.23 Å) do not depart from that in 4, and even the ferrocene units remain undistorted (tilt angles <2°).

### Reactions of (chlorocarbonyl)ferrocene with germenolates 2 and 3

**Scheme 3:**
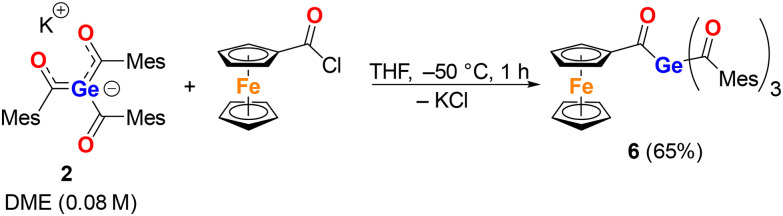
Synthesis of tetraacylgermane 6 (Mes = mesityl).

The next objective was to use germenolate 2 as a nucleophile for the formation of mixed tetraacylgermanes. Therefore, 2 was dissolved in DME, and the solution was added to an equimolar amount of (chlorocarbonyl)ferrocene dissolved in THF at −50 °C. After aqueous workup and recrystallisation from *n*-pentane, compound 6 was isolated in a 65% yield (Scheme 3).

**Fig. 3:**
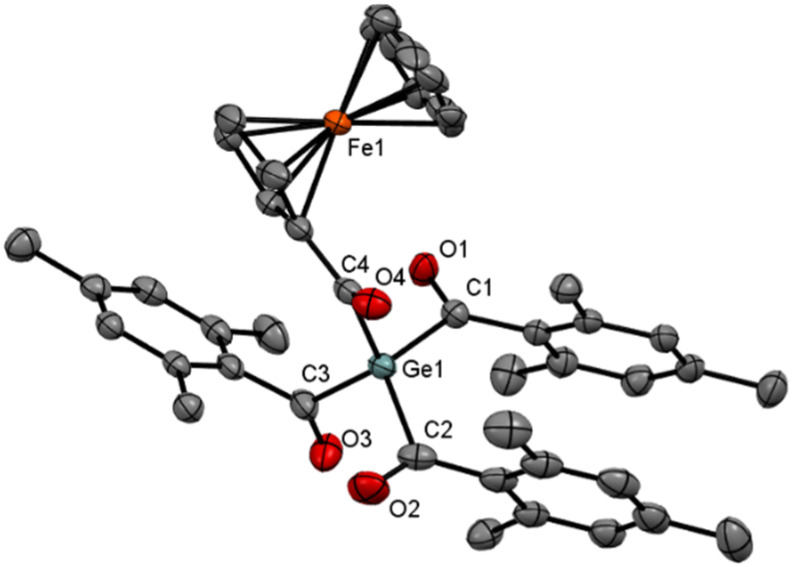
ORTEP representation of compound 6. Thermal ellipsoids are depicted at the 50% probability level. Hydrogen atoms and alternative positions of the disordered atoms are omitted for clarity. Selected bond lengths (Å) and bond angles (°) with estimated standard deviations: Ge(1)–C(1) 2.0530(16), Ge(1)–C(2) 2.0850(18), Ge(1)–C(3) 2.0415(17), Ge(1)–C(4) 2.0278(16), O(1)–C(1) 1.2147(19), O(2)–C(2) 1.2110(2), O(3)–C(3) 1.221(6), O(4)–C(4) 1.223(2), C(1)–C(5) 1.497(2), C(2)–C(14) 1.499(3), C(3)–C(23) 1.490(2), C(4)–C(32) 1.458(2).

The ^13^C NMR spectrum of compound 7 shows two different carbonyl signals at *δ*_C_ 221.4 and 233.1, attributable to FcC(O) and MesC(O) groups, respectively. Crystallization from acetone at room temperature produced single crystals that were used for X-ray diffraction analysis. The molecular structure is shown in Fig. 3. The Ge–C distances in the molecule of 7 are 2.0254(16), 2.0285(18), 2.0452(19) Å for the mesitoyl groups and 2.0278(17) Å for the ferrocenylcarbonyl substituent; the associated C–Ge–C angles are 99–116°. Two of the mesityl groups are engaged in a structure-stabilizing π⋯π stacking interaction (ring centroid distance: 3.8056(10) Å; dihedral angle of the benzene rings: 3.96(8)°). No similar interaction is detected between the third mesitoyl group and the substituted cyclopentadienyl ring.

**Scheme 4:**
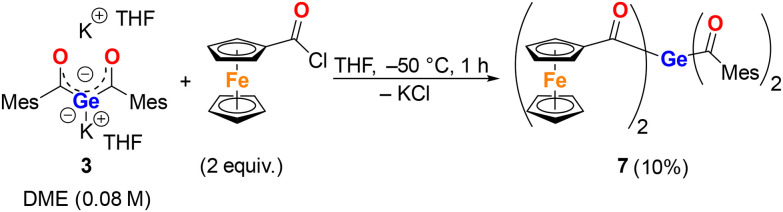
The reaction of 3 with FcC(O)Cl leading to compound 7.

In the same vein, we employed diacylgermenolate 3 as the nucleophile. This compound was dissolved in THF, and the solution was added to (chlorocarbonyl)ferrocene (2 equiv.) dissolved in THF at −50 °C. The reaction proceeded less satisfactorily than the analogous reaction with 2. After aqueous workup and preparative thin-layer chromatography, it afforded the targeted compound 7 as a red oil in only 10% yield (Scheme 4). The NMR spectra of 7 displayed all expected signals; the characteristic ^13^C NMR resonances due to the C=O groups were detected at *δ*_C_ 233.1 and 221.5.

### Photochemical investigations

**Fig. 4:**
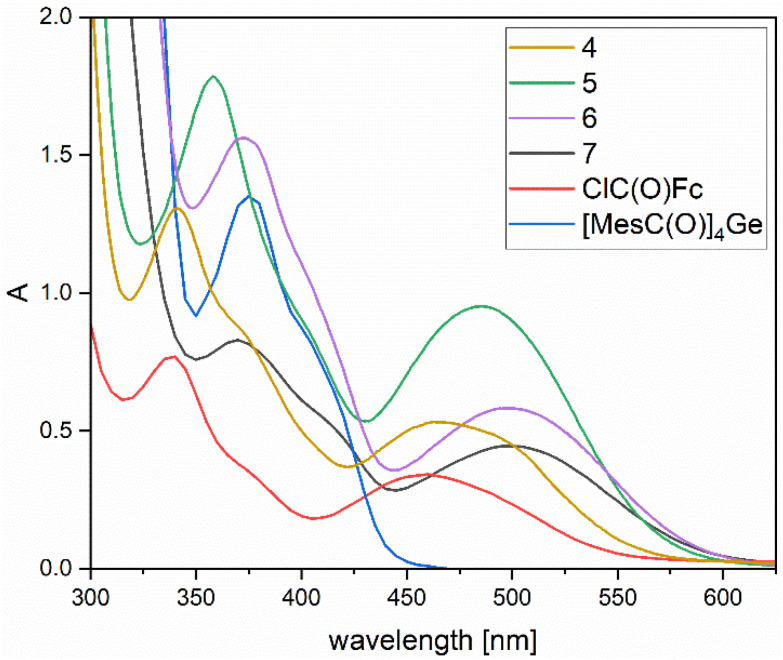
UV-Vis absorption spectra of 4–7, (chlorocarbonyl)ferrocene and Ge(C(O)Mes)_4_ (CH_2_Cl_2_, *c* = 5 × 10^−4^ mol L^−1^).

For envisioned photochemical investigations, we initially focused on examining the UV-Vis absorption spectra of the compounds and compared them with the spectra of (chlorocarbonyl)ferrocene [FcC(O)Cl] and tetramesitoyl-germane [(MesC(O))_4_Ge] to assess their potential for light absorption and photoinitiation. The UV/Vis spectra are shown in Fig. 4.

The longest wavelength absorption bands, around 460–502 nm, can be assigned to d → d transitions of the ferrocene unit.^20^ The second band in the visible region, centered around 340–426 nm, corresponds to n/σ → π* transitions originating from the carbonyl lone pair and the Ge–C σ-bond of the acyl moieties. Interestingly, the mixed acyl derivatives 6 and 7 exhibit a clear bathochromic shift of both bands.

Based on the absorption behavior, we irradiated compounds 4–7 at four different wavelengths (*λ* = 365, 405, 550, and 590 nm). The ferrocenoyl-substituted derivatives 4 and 5 did not show any detectable photochemical reactivity. This observation is consistent with previous findings by Pannell and co-workers, who reported similar results for ferrocenoyl-substituted silanes.^21^ As noted in their work, the ferrocene moiety acts as a triplet quencher^22^ and thus suppresses the α-cleavage pathway.

**Scheme 5:**
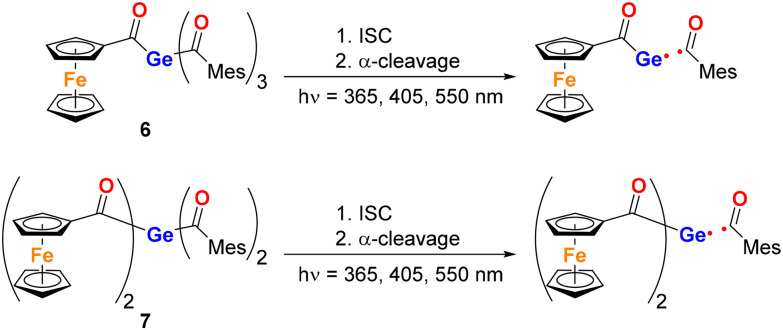
Photochemical reactivity of 6 and 7.

In stark contrast, compounds 6 and 7 underwent the characteristic α-cleavage of the mesitoyl moiety upon irradiation at 365 nm, 405 nm and, notably, also at 550 nm. This represents a significant finding, as it marks the highest wavelength reported to date for inducing α-cleavage in acylgermanes. The ability of these compounds to undergo efficient bond homolysis at such low-energy visible light highlights their potential for applications in photopolymerization and light-triggered processes under mild conditions (Scheme 5). The conversion was monitored by ^1^H NMR spectroscopy. The obtained spectra can be found in the SI (Fig. S16). Here, polarized ^1^H resonances at *δ*_H_ ≈ 10 indicate that hydrogen atom transfer from the benzoyl radical led to the formation of the corresponding aldehyde.

Accordingly, we have performed an exemplary quantum-yield determination for the light-induced α-cleavage of 6 under LED-irradiation, following established procedures.^23^ Here, LED wavelength between 385 nm and 500 nm were chosen to match both the π → π* (ferrocene) and n/σ → π* (acyl) absorption bands. The results are shown in Table 1. Within the experimental timeframe, significant bleaching is observed only for light-irradiation below 400 nm. This illustrates, that efficient α-cleavage of 6 is strongly linked to the overlap of the LED-emission with the n/σ → π* acyl absorbance. The quantum yields are approximately one order of magnitude smaller than what is reported for the commonly used bisacylgermane photoinitiator Ivocerin® (0.83 ± 0.01).^4^ Optical spectra and a detailed description of the experimental procedure are available in the SI.

**Table 1:** Quantum yields *ϕ* and extinction coefficients *ε* of **6** in THF at the given wavelength *λ*

*λ*/nm	*ϕ*	*ε*/L mol^−1^ cm^−1^
385	3.0 ± 0.2 × 10^−2^	2.45 ± 0.01 × 10^3^
405	2.4 ± 0.2 × 10^−2^	1.99 ± 0.01 × 10^3^
450	—	7.32 ± 0.07 × 10^2^
500	—	9.97 ± 0.12 × 10^2^

### Si analogous compounds

**Scheme 6:**
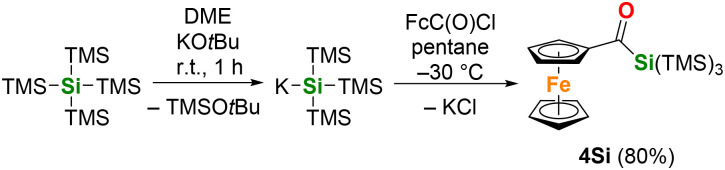
Synthesis of acylsilane 4Si.

Based on these results, we also wanted to briefly investigate the photochemical behavior of the related silicon compounds. The Si analogue of the acylgermane 4, was synthesized from the reaction of *in situ*-generated KSi(SiMe_3_)_3_ and (chlorocarbonyl)ferrocene. Recrystallization in acetone afforded 4Si in an 80% yield (Scheme 6).

**Fig. 5:**
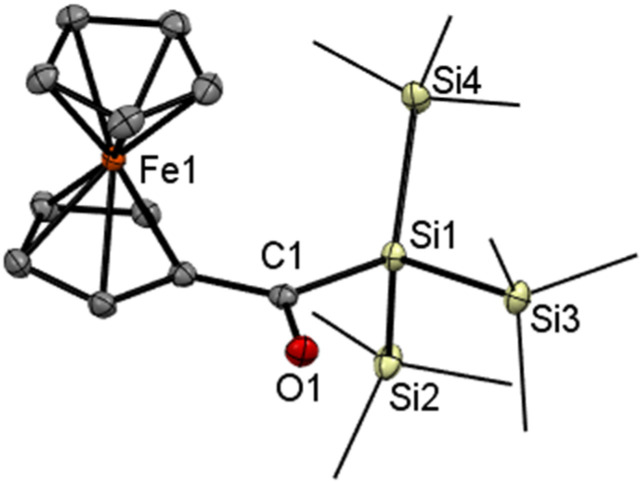
ORTEP representation of compound 4Si. Thermal ellipsoids are depicted at the 50% probability level. Hydrogen atoms are omitted for clarity. Selected bond lengths (Å) and bond angles (°) with estimated standard deviations: Si(1)–C(1) 1.9479(12), Si(1)–Si(2) 2.3546(5), Si(1)–Si(3) 2.3582(4), Si(1)–Si(4) 2.3578(4), C(1)–O(1) 1.2337(14), C(1)–C(7) 1.4723(16).

Compound 4Si was characterized by ^1^H and ^13^C NMR spectroscopy. A typical, downfield-shifted carbonyl signal at *δ*_C_ 234.1 was observed in the ^13^C NMR spectrum. Crystals suitable for X-ray diffraction were obtained by cooling a concentrated acetone solution of compound 4Si to −30 °C. The molecular structure is shown in Fig. 5. Compound 4Si crystallizes isostructural with its germanium analog. While the C1=O1 bond length of 4Si (1.2336(15) Å) does not practically differ from that in 4, the C1–Si1 bond is expectedly shorter (1.9479(13) Å) than the corresponding C–Ge bond in 4. The ferrocene unit shows parallel cyclopentadienyl rings (tilt angle: 3.52(7)°), and Fe–C distances in the 2.0383(12)–2.0570(12) Å range.

Similar to 4 and 5, compound 4Si showed no photochemical reactivity. Even so, the attempted synthesis of tetrakis(ferrocenylcarbonyl)silane, as well as the Si-analogous compounds to 7 and 8, was not successful.

**Scheme 7:**
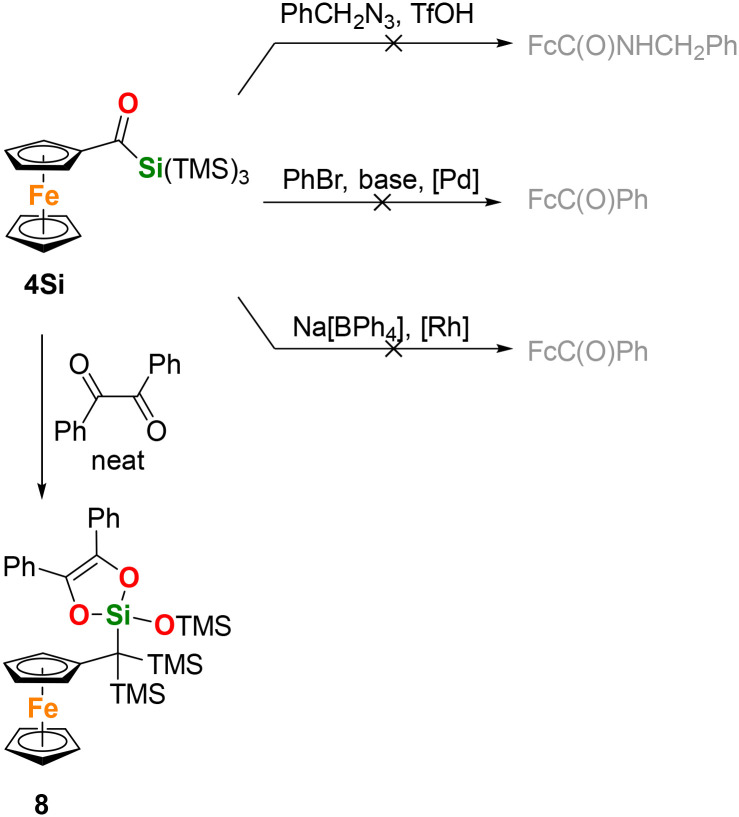
Reaction tests with 4Si (see text for details).

Representative compounds 4 and 4Si were further investigated for their reactivity in the selected C–C bond-forming reactions reported for acylsilanes (Scheme 7). Thus, following the literature procedure,^24^ compound 4Si was reacted with benzyl azide (1.8 equiv.) in the presence of triflic acid (2.0 equiv.) in dichloromethane (*c*(4Si) = 50 mM) at room temperature for 15 min. After evaporation and chromatography, 10% of 4Si was recovered along with a mixture of unidentified ferrocene compounds. The desired amide was not detected.

**Fig. 6:**
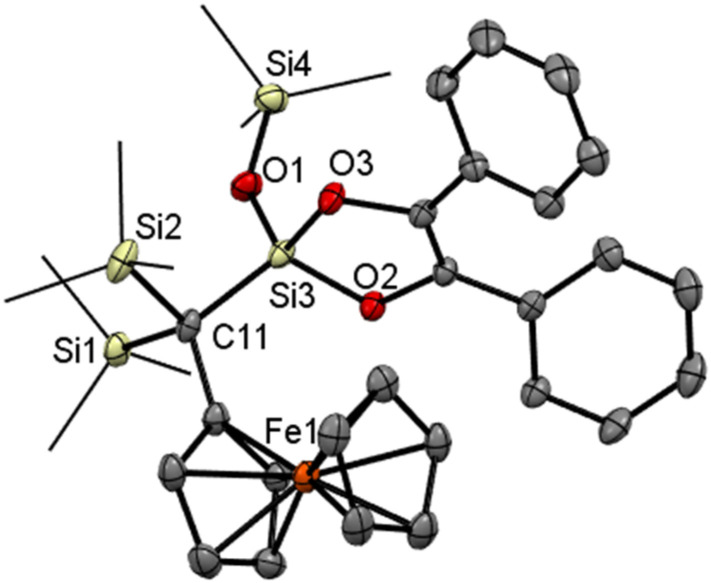
ORTEP representation of compound 8. Thermal ellipsoids are depicted at the 50% probability level. Hydrogen atoms are omitted for clarity. Selected bond lengths (Å) and bond angles (°) with estimated standard deviations: Si(3)–O(1) 1.6053(19), Si(3)–O(2) 1.6659(18), Si(3)–O(3) 1.6668(19), Si(3)–C(11) 1.844(3), Si(1)–C(11) 1.9360(3), Si(2)–C(11) 1.9190(3), Si(4)–O(1) 1.6500(2).

Next, we focused on converting 4Si into ketones.^25^ To achieve this, we treated 4Si (1.5 equiv.) with bromobenzene (1.0 equiv.) and K_3_PO_4_ (2.0 equiv.) in wet THF (6 equiv. of water) in the presence of 2 mol% of methanesulfonato(triphenylphosphine)(2′-amino-1,1′-biphenyl-2-yl-κ^2^*C*,*N*)palladium(ii) at 60 °C for 24 h. From the resulting mixture, we only isolated ferrocenecarboxaldehyde (33%), formally the product of C(O)–Si bond cleavage, and unidentified ferrocene derivatives. Benzoylferrocene and the starting material were not detected. A reaction of 4Si with sodium tetraphenylborate (1.5 equiv.), performed in the presence of [Rh(μ-Cl)(cod)]_2_ (6 mol% Rh, cod = cycloocta-1,5-diene) in *p*-xylene (*c*(4Si) = 25 mM, 130 °C for 24 hours),^26^ proceeded similarly, yielding ferrocenecarboxaldehyde (49%) and a mixture of monosubstituted ferrocenes after workup. Benzoylferrocene and 4Si were not detected again. The limited reactivity of 4Si was tentatively attributed to the changed silane substituent, whose steric bulk and electronic properties render the C(O)–Si bond less reactive. Lastly, compound 4Si was mixed with benzil (1.05 equiv.) and heated without solvent to 140 °C for 16 h.^27^ Subsequent chromatography yielded 8 as the only product (98% yield). The compound was characterized spectroscopically, and its molecular structure was confirmed by single-crystal X-ray diffraction analysis (Fig. 6).

The formation of 8 could be rationalized as [4 + 2] cycloaddition of *in situ*-generated silene ((Me_3_Si)_2_C=C(Fc)(OSiMe_3_) in the present case) with benzil and subsequent, thermally-induced isomerization of dioxasilacyclohexene intermediate, ultimately leading to dioxasilacyclopentene 6, as reported.^27^ Notably, a similar reaction with 4 did not proceed.

Compound 4Si crystallizes with two crystallographically independent molecules that differ only by the orientation of the methyl groups in the OSiMe_3_ arm. For steric reasons, the pivotal bond C1–C11 is deflected from the parent cyclopentadienyl plane (by approximately 12°) and its substituents are distributed so that one SiMe_3_ group and the C_2_O_2_Si ring are oriented toward the ferrocene unit while the remaining SiMe_3_ groups points away from it. The C_2_O_2_Si ring is planar with distances corresponding to C–O single (1.40 Å) and C=C double bonds (1.35 and 1.38 Å).

### Electrochemistry

Considering the presence of the redox-active ferrocenyl groups, we also studied the electrochemical behavior of the prepared compounds using cyclic voltammetry at a glassy carbon disc electrode in dichloromethane containing 0.1 M Bu_4_N[PF_6_] as the supporting electrolyte. The multiferrocenyl systems were also studied by differential pulse voltammetry (DPV) under identical conditions. The potentials are quoted relative to the ferrocene/ferrocenium reference^28^ (see Experimental).

**Fig. 7:**
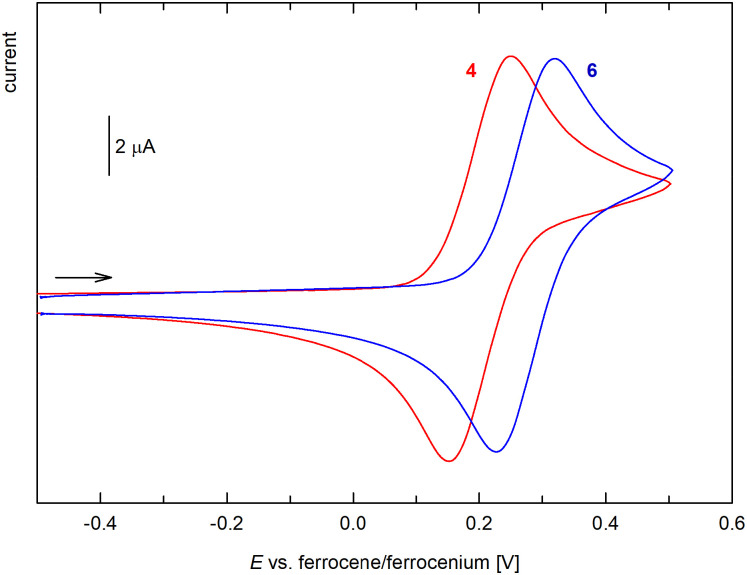
Cyclic voltammograms of 4 and 6 (CH_2_Cl_2_, 0.1 M Bu_4_N[PF_6_], glassy carbon electrode, 100 mV s^−1^ scan rate; the scan rate is indicated with an arrow). The cyclic voltammogram of 4Si is virtually identical with that of compound 4 and, therefore, is not presented here.

In the accessible potential range, compound 4 and 4Si showed a single reversible redox transition (Fig. 7) attributable to the ferrocene/ferrocenium couple. The formal redox potentials determined as the average of the anodic and cathodic peak currents, *E*°′ = ½(*E*_pa_ + *E*_pc_), were 0.21 V for both compounds, indicating that the oxidation of the ferrocene units in these compounds is more difficult than in ferrocene itself due to the overall electron-withdrawing effect of the acyl moiety.

**Fig. 8:**
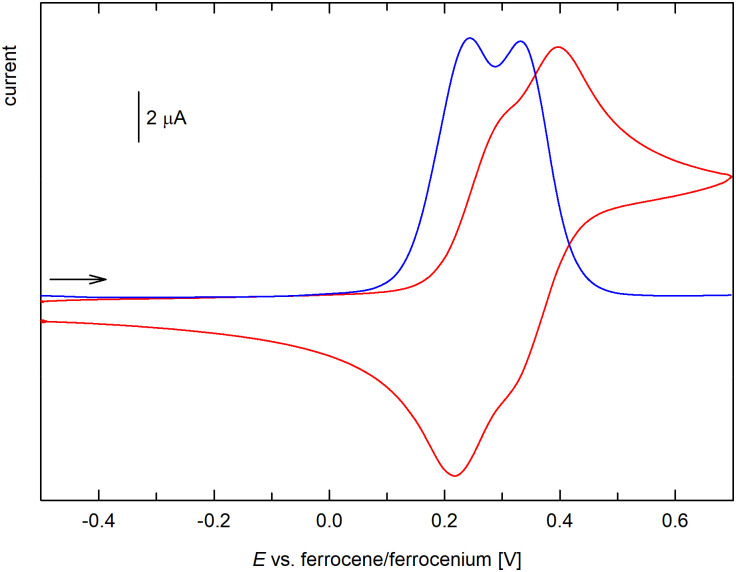
Cyclic (red) and differential pulse voltammogram (blue) of 7 (CH_2_Cl_2_, 0.1 M Bu_4_N[PF_6_], glassy carbon electrode; cyclic voltammogram: 100 mV s^−1^ scan rate; DPV: step 1 mV, pulse width 50 mV; the scan rate is indicated with an arrow).

Similar behavior was observed for compound 6 (Fig. 7), which also displayed one reversible oxidation at a slightly higher potential, 0.28 V, most likely due to a cumulative influence of the acyl substituents at the Ge atom. Conversely, the bis(ferrocenylcarbonyl) derivative 7 underwent two closely separated, reversible redox changes attributable to successive oxidation of the ferrocenyl groups (Fig. 8). The two-step behavior was confirmed by differential pulse voltammetry, which showed two separated oxidation peaks of equal intensity. The stepwise oxidation suggests an electronic communication between the ferrocene units in 7, when the first electron removal affects the subsequent oxidation (the first oxidation not only generates a positively charged species but also converts the strongly electron-donating ferrocenyl group into an electron-withdrawing ferrocenium; *cf*. the Hammett *σ*_p_ constants of ferrocenyl and the corresponding ferrocenium of −0.18 and 0.29, respectively).^29^ The redox potentials determined for the two redox steps from DPV were 0.26 and 0.35 V *vs*. the ferrocene reference.^30^ Similar behavior and comparable separation of the two oxidation steps was reported for diferrocenylmethane (Fc_2_CH_2_, 120 mV)^31^ and silane Fc_2_SiMe_2_ (150 mV).^32^ The related compounds [(C_5_Me_5_)Fe(C_5_H_4_–Y–C_5_H_4_)Fe(C_5_Me_5_)], where Y = CMe_2_, SiMe_2_, and GeMe_2_, showed smaller potential difference, decreasing from CMe_2_ (113 mV) through SiMe_2_ (93 mV) to GeMe_2_ (74 mV) spacer.^33^

**Fig. 9:**
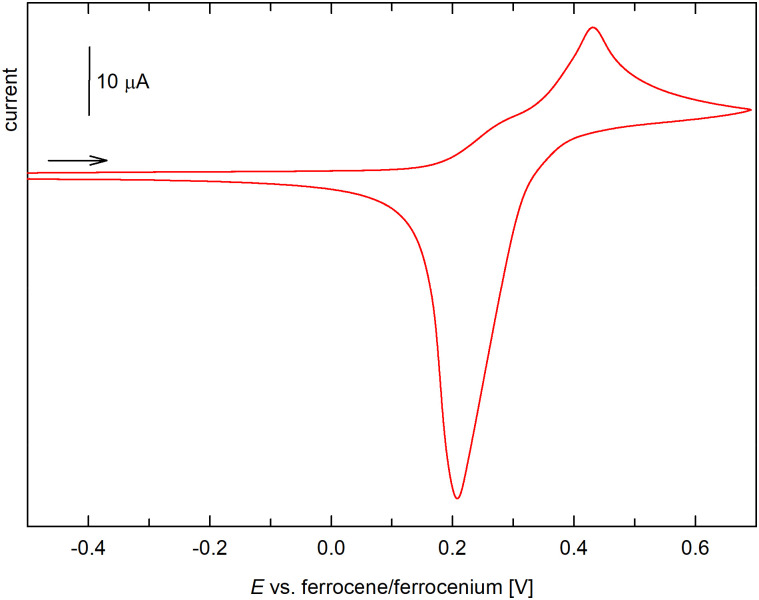
Cyclic voltammograms of 5 (CH_2_Cl_2_, 0.1 M Bu_4_N[PF_6_], glassy carbon electrode, 100 mV s^−1^ scan rate; the scan rate is indicated with an arrow).

**Fig. 10:**
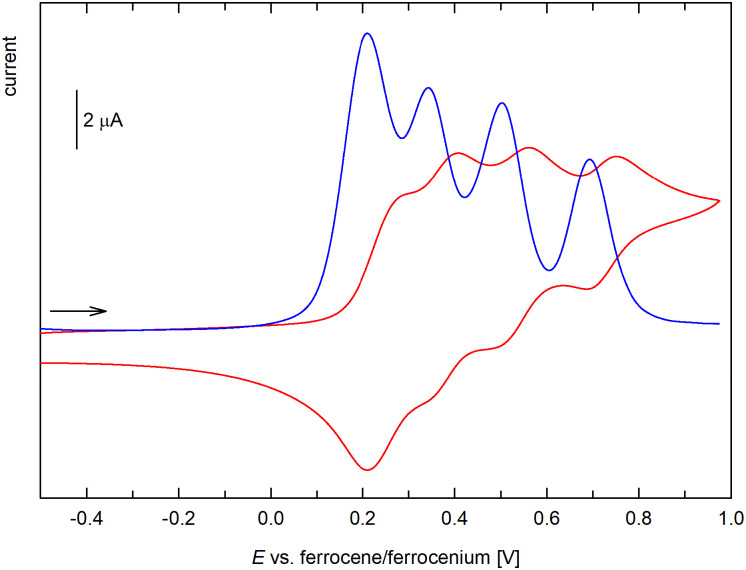
Cyclic (red) and differential pulse voltammogram (blue) of 5 (CH_2_Cl_2_, 0.1 M Bu_4_N[B(C_6_F_5_)_4_], glassy carbon electrode; cyclic voltammogram: 100 mV s^−1^ scan rate; DPV: step 1 mV, pulse width 50 mV; the scan rate is indicated with an arrow).

The redox response of tetraferrocenyl derivative 5 was the most complicated (Fig. 9). In dichloromethane/Bu_4_N[PF_6_], the compound displayed two consecutive oxidations (peaks at ≈0.29 and 0.43 V). During the reverse scan, however, only an intense stripping peak at approximately 0.2 V was observed, suggesting adsorption of the electrochemically generated (cationic) species on the electrode surface. When the supporting electrolyte was changed for Bu_4_N[B(C_6_F_5_)_4_],^34^ the response simplified and four distinct oxidation steps extending over a relatively wider potential range were detected in both the cyclic voltammogram and DPV (Fig. 10), resulting from successive and reversible oxidation of the ferrocenyl groups. The *E*°′ values for the individual oxidation steps obtained from the DPV data were 0.24, 0.37, 0.53, and 0.72 V.

## Conclusions

In summary, we report that metathesis reactions of (chlorocarbonyl)ferrocene with potassium germanides and germenolates formed *in situ* from KO*t*Bu and Ge(SiMe_3_)_4_ or Ge(C(O)Mes)_4_ produce various α-germyl ferrocenyl ketones. Compounds containing one, two, or four ferrocenyl groups were isolated and studied using a combination of spectroscopic methods, single-crystal X-ray diffraction analysis, and voltammetric techniques. Photochemical studies revealed distinct behavior depending on the substitution pattern: purely ferrocenoyl-substituted derivatives (4, 5) were photoinert due to triplet quenching by the ferrocene moiety, whereas mixed ferrocenoyl/mesitoyl compounds (6, 7) underwent α-cleavage upon visible light irradiation. Remarkably, these compounds displayed photoreactivity up to 550 nm, the longest wavelength reported for α-cleavage in acylgermanes to date, underscoring their potential for mild, visible-light-driven processes. Additionally, the synthesis and reactivity of the silicon analogue 4Si were explored. While structurally analogous to its germanium counterpart, 4Si showed similarly limited photoreactivity. However, a high-yielding formal [4 + 2] cycloaddition with benzil afforded a siladioxacyclopentene product 8, demonstrating that alternative reactivity pathways are accessible *via* thermal activation.

Finally, our results establish ferrocenyl-substituted germyl and silyl ketones as modular platforms that combine redox and photochemical functionality. Their tunable light absorption, redox properties, and structural diversity provide a foundation for the future development of photoredox-responsive materials, redox sensors, and light-triggered polymerization systems.

## Experimental section

### Materials and methods

All experiments were performed under a nitrogen atmosphere using standard Schlenk techniques. Solvents were dried using a column solvent purification system.^35^ Me_3_SiCl (≥99%), GeCl_4_ (99.99%), KO*t*Bu (>98%), THF-d_8_ (99.5 atom% D), chloroform-d (99.8 atom% D) and benzene-d_6_ (99.5 atom% D) were used without any further purification. For the measurement of air-sensitive samples, benzene-d_6_ and THF-d_8_ were additionally dried above a sodium/potassium alloy under 12-hour reflux. Likewise, chloroform-d was dried over activated 4 Å molecular sieves. Tetrakis(trimethylsilyl)germane,^15^ tetramesitoylgermane,^3^ tris(2,4,6-trimethylbenzoyl)germenolate (2),^5^ and dipotassium bis(2,4,6-trimethylbenzoyl)bisgermenolate (3),^6^ FcC(O)Cl,^36^ and Bu_4_N[B(C_6_F_5_)_4_]^37^ were prepared according to published procedures.^3^ Melting points were determined using the Stuart SMP50 apparatus and are uncorrected. Elemental analyses were carried out on a Hanau Vario Elementar EL apparatus.

UV-Vis spectra were acquired either using a TIDAS UV-Vis spectrometer equipped with optical fibers and a 1024-pixel diode-array detector (J&M Analytik AG, Essingen, Germany), or a PerkinElmer Lambda 5 spectrometer. FTIR spectra were obtained on a Bruker α-P Diamond ATR Spectrometer running OPUS 7.5 software in transmission mode from the solid samples.

^1^H, ^13^C, and ^29^Si NMR spectra were recorded either on a Varian INOVA 400, a 200 MHz Bruker AVANCE DPX, or a 400 MHz Jeol JNM-ECZL spectrometer with Royal HFX-Probes and an autosampler. The chemical shifts (*δ* in ppm) are referenced *versus* tetramethysilane using the internal ^2^H-lock signal of the solvent. Details of structure determination by single-crystal X-ray diffraction analysis are available in the SI.

Electrochemical measurements were performed at room temperature using a computer-controlled multipurpose μAUTOLAB III instrument (Eco Chemie, the Netherlands) and a Metrohm three-electrode cell equipped with a glassy carbon disc (2 mm diameter) working electrode, a platinum sheet auxiliary electrode, and an Ag/AgCl (3 M KCl) reference electrode. The samples were dissolved in anhydrous dichloromethane to give a solution containing approximately 1 mM of the analyzed compound and 0.10 M Bu_4_N[PF_6_] (TCI) or Bu_4_N[B(C_6_F_5_)_4_] as the supporting electrolyte. The solutions were deaerated by bubbling with argon before the measurement and then kept under an argon flow. Decamethylferrocene was added as an internal standard for the final scans, and the potentials were recalculated to the ferrocene/ferrocenium scale by subtracting 0.548 V (for Bu_4_N[PF_6_]) and 0.614 V (for Bu_4_N[B(C_6_F_5_)_4_]).^34^

### Syntheses

#### Synthesis of 4

A solution of (Me_3_Si)_3_GeK (solution a), was freshly prepared from Ge(SiMe_3_)_4_ (1; 1.00 g, 2.73 mmol) and KO*t*Bu (0.33 g, 2.73 mmol) in 40 mL of 1,2-dimethoxyethane (DME). Solution b was prepared separately by dissolving (chlorocarbonyl)ferrocene (0.68 g, 2.73 mmol) in 40 mL THF. Solution a was introduced into one feed line and solution b into a second feed line of a continuous flow reactor (Syrris Asia reciprocating syringe pump). The two solutions were pumped simultaneously at a total flow rate of 18 mL min^−1^ (9 mL min^−1^ per solution) and mixed *via* a Y-mixer before entering the reactor coil (PTFE tubing, inner diameter 0.8 mm, volume 3.07 mL; for details, see SI). The reaction was conducted at −50 °C under a nitrogen atmosphere. After a residence time of 10.2 seconds, the reaction mixture exited the reactor and was collected into a saturated aqueous NH_4_Cl solution, and the organic layer was separated. The aqueous phase was washed with dichloromethane (3 × 50 mL), and the combined organic layers were dried with Na_2_SO_4_ and evaporated in vacuum. The crude product was purified by flash column chromatography (silica gel, Et_2_O). Recrystallisation from acetone gave dark red crystals of 4. Yield: 0.44 g (51%).

M.p. 85–87 °C. Anal. calc. for C_20_H_36_FeGeOSi_3_: C, 47.55; H, 7.18%. Found: C, 48.08; H, 7.03%. ^13^C{^1^H} NMR (C_6_D_6_): *δ* 232.10 (*C*=O), 89.33, 71.17 and 69.89 (*C*_5_H_4_), 69.70 (*C*_5_H_5_), 2.56 (Si(*C*H_3_)_3_). ^1^H NMR (C_6_D_6_). *δ* 4.75 (t, *J*′ = 1.9 Hz, 2H, C_5_*H*_4_), 4.07 (t, *J*′ = 1.9 Hz, 2H, C_5_*H*_4_), 4.06 (s, 5H, C_5_*H*_5_), 0.40 (s, 27H, Si(C*H*_3_)_3_). ^29^Si INEPT (C_6_D_6_): *δ* −5.10. FTIR (neat): *ν*(C=O) 1584 cm^−1^ UV-Vis: *λ* [nm] (*ε* [L mol^−1^ cm^−1^]) = 342 (2604), 361 (1891), 468 (1051).

#### Synthesis of 5

A solution of (Me_3_Si)_3_GeK in 25 mL of DME, freshly prepared from 1 (1.00 g, 2.73 mmol) and KO*t*Bu (0.33 g, 2.73 mmol) in 25 mL of DME, was slowly added to a solution of (chlorocarbonyl)ferrocene (2.71 g, 10.92 mmol) and an excess KF (2.0 g, 34.4 mmol) in 25 mL Et_2_O at −30 °C. The solution was slowly warmed up to room temperature and stirred for 12 h. After aqueous workup with saturated NH_4_Cl solution, the organic layer was separated, dried over Na_2_SO_4_, and concentrated under reduced pressure. The product was recrystallized from acetone, giving dark red crystals of 5. Yield: 1.90 g (75%).

M.p. 250–252 °C. Anal. calc. for C_44_H_36_Fe_4_GeO_4_: C, 57.15; H, 3.92%. Found: C, 57.12; H, 3.89%. ^13^C{^1^H} NMR (CDCl_3_): *δ* 222.31 (*C*=O), 87.17, 73.72 and 70.01 (*C*_5_H_4_), 70.22 (*C*_5_H_5_). ^1^H NMR (CDCl_3_): *δ* 5.03 (s, 8H, C_5_*H*_4_), 4.63 (s, 8H, C_5_*H*_4_). 4.23 (s, 20 H, C_5_*H*_5_). IR (neat): *ν*(C=O) 1575 cm^−1^. UV-Vis: *λ* [nm] (*ε*[L mol^−1^ cm^−1^]) = 359 (3569), 389 (2112), 485 (1902).

#### Synthesis of 6

Potassium tris(2,4,6-trimethylbenzoyl)germanide·0.5DME (1.00 g, 1.67 mmol) was dissolved in 20 mL DME and added to a solution of (chlorocarbonyl)ferrocene (0.41 g, 1.67 mmol) in 20 mL THF at −50 °C. Subsequently, the reaction mixture was brought to room temperature and stirred for 1 h. The solution was then added to 200 mL of saturated NH_4_Cl solution. The organic layer was separated, and the aqueous phase was washed with dichloromethane (3 × 100 mL). The combined organic layers were dried with Na_2_SO_4_ and evaporated under vacuum. The residue was recrystallized from *n*-pentane, giving red crystals of 6. Yield: 0.79 g (65%).

M.p. 173–175 °C. Anal. calc. for C_41_H_42_FeGeO_4_: C, 67.71; H, 5.82%. Found: C, 67.69; H, 5.83%. ^13^C{^1^H} NMR (C_6_D_6_): *δ* 233.39 (Mes*C*=O), 221.71 (Fc*C*=O), 142.38, 139.39, 133.58 and 129.15 (Mes–*C*), 86.02, 72.42 and 70.69 (*C*_5_H_4_), 70.23 (*C*_5_H_5_), 21.08 and 19.73 (Mes–*C*H_3_). ^1^H NMR (C_6_D_6_): *δ* 6.40 (s, 6H, Mes–*H*), 5.01 (t, *J*′ = 1.9 Hz, 2H, C_5_*H*_4_), 4.03 (t, *J*′ = 1.9 Hz, 2H, C_5_*H*_4_), 4.00 (s, 5H, C_5_*H*_5_), 2.34 (s, 18H, Mes–C*H*_3_), 1.97 (s, 9H, Mes–C*H*_3_). IR (neat): *ν*(C=O) 1653, 1637, 1607, 1586 cm^−1^. UV-Vis: *λ* [nm] (*ε* [L mol^−1^ cm^−1^]) = 373 (3410), 393 (2682), 500 (1292).

#### Synthesis of 7

K_2_Ge[(CO)Mes]_2_·2THF (1.10 g, 1.86 mmol) was dissolved in 20 mL THF and added to a solution of (chlorocarbonyl)ferrocene (0.92 g, 3.72 mmol) in 20 mL THF at −50 °C. Subsequently, the reaction mixture was brought to room temperature and stirred for 1 h. The solution was then added to a saturated NH_4_Cl solution (200 mL). After phase separation, the aqueous phase was washed three times with 100 mL of dichloromethane, and the combined organic layers were dried over Na_2_SO_4_ and then evaporated under vacuum. The crude product was purified by preparative thin-layer chromatography (silica gel, toluene/Et_2_O; 40 : 1), giving 7 as a red oil. Yield: 0.16 g (10%).

Anal. calc. for C_41_H_39_Fe_2_GeO_4_: C, 57.75; H, 4.61%. Found: C, 57.72; H, 4.59%. ^13^C{^1^H} NMR (C_6_D_6_): *δ* 233.14 (Mes*C*=O), 221.49 (Fc*C*=O), 142.26, 139.30, 133.64 and 129.17 (Mes–*C*), 86.23, 72.83 and 70.85 (*C*_5_H_4_), 70.44 (*C*_5_H_5_), 21.10 and 19.87 (Mes–*C*H_3_). ^1^H-NMR (C_6_D_6_): *δ* 6.39 (s, 3H, Mes–*H*), 5.91 (t, *J*′ = 1.9 Hz, 4H, C_5_*H*_4_), 4.07 (t, *J*′ = 1.9 Hz, 4H, C_5_*H*_4_), 4.04 (s, 10H, C_5_*H*_5_), 2.39 (s, 12H, Mes–C*H*_3_), 1.96 (s, 6H, Mes–C*H*_3_), IR (neat): *ν*(C=O) 1640, 1608, 1591 cm^−1^. UV-Vis: *λ* [nm] (*ε* [L mol^−1^ cm^−1^]) = 370 (1648), 494 (882).

#### Synthesis of 4Si

A solution of (Me_3_Si)_3_SiK in 25 mL of DME was freshly prepared from Si(SiMe_3_)_4_ (5.50 g, 17.18 mmol) and KO*t*Bu (1.92 g, 17.18 mmol) and slowly added to a solution of (chlorocarbonyl)ferrocene (4.25 g, 17.18 mmol) in 25 mL *n*-pentane at −30 °C. The solution was slowly warmed up to room temperature and stirred for an additional 1 h. After aqueous workup with saturated NH_4_Cl solution, the organic layer was separated, dried over Na_2_SO_4_, and the solvents evaporated under reduced pressure. The product was purified *via* flash column chromatography using diethyl ether and recrystallized from acetone, giving dark red crystals of 1. Yield: 6.62 g (80%).

M.p. 90–92 °C. Anal. calc. for C_20_H_36_FeOSi_4_: C, 52.74; H, 7.59%. Found: C, 52.77; H, 7.63% ^13^C{^1^H} NMR (C_6_D_6_): *δ* 234.14 (*C*=O), 89.50, 71.23, 69.78 (C_5_H_4_), 69.72 (C_5_H_5_), 1.98 (Si(CH_3_)_3_). ^1^H NMR (C_6_D_6_): *δ* 4.77 (t, *J*′ = 1.9 Hz, 2H, C_5_H_4_), 4.07 (t, *J*′ = 1.9 Hz, 2H, C_5_H_4_); 4.05 (s, 5H, C_5_H_5_), 0.37 (s, 27H, Si(CH_3_)_3_). ^29^Si INEPT (C_6_D_6_): *δ* −11.45 (Si–(*Si*Me_3_)_3_) −71.43 (*Si*–(SiMe_3_)_3_). IR (neat): *ν*(C=O) 1564 cm^−1^. UV-Vis: *λ* [nm] (*ε*[L mol^−1^ cm^−1^]) = 343 (2740), 460 (1270).

#### Reaction of 4Si with benzil

Compound 4Si (46.1 mg, 0.100 mmol) was mixed with benzil (22.1 mg, 0.105 mmol) in a dry tube. The tube was sealed and heated at 140 °C for 16 hours. After cooling, the residue was purified by chromatography over a silica gel column, eluting with dichloromethane–methanol (20 : 1). A single orange band was evaporated, leaving 8 as an orange solid. Yield: 66.0 mg (98%). The crystal used for structure determination was grown from methanol at −18 °C.

M.p. 125.5–126.5 °C. Anal. calc. for C_34_H_46_FeSi_4_O_3_: C, 60.87; H, 6.91%. Found: C, 60.80; H, 6.84%.^13^C{^1^H} NMR (CDCl_3_): *δ* 1377.11 (C–Ph), 133.51 (C^ipso^ Ph), 128.34 (*m*-CH Ph), 127.62 (*p*-CH Ph), 127.25 (*o*-CH Ph), 88.38 (C^ipso^ C_5_H_4_), 69.63 (CH C_5_H_4_), 69.32 (C_5_H_5_), 66.61 (CH C_5_H_4_), 12.50 (*C*–Fc), 2.30 (SiMe_3_), 1.77 (OSiMe_3_). ^1^H NMR (CDCl_3_): *δ* 7.55–7.58 (m, 4 H, Ph), 7.28–7.31 (m, 6 H, Ph), 4.16 (s, 5 H, C_5_H_5_), 4.15 (t, *J*′ = 1.9 Hz, 2 H, CH C_5_H_4_), 4.13 (vt, *J*′ = 1.9 Hz, 2 H, CH C_5_H_4_), 0.25 (s, 18 H, SiMe_3_), 0.22 (s, 9 H, OSiMe_3_), ^29^Si{^1^H} NMR (CDCl_3_): *δ* 13.61 (OSiMe_3_), 1.92 (2 Si, CSiMe_3_), −35.60 (OSi). HRMS calc. for C_34_H_46_FeSi_4_O_3_ (M^+^): 670.1874, found 670.1893.

## Conflicts of interest

There are no conflicts to declare.

## Supplementary Material

DT-054-D5DT02029H-s001

DT-054-D5DT02029H-s002

## Data Availability

The data supporting this article have been included as part of the supplementary information (SI). Supplementary information: details of structure determination, additional structure diagrams, copies of the NMR spectra. See DOI: https://doi.org/10.1039/d5dt02029h. CCDC 2478328–2478332 contain the supplementary crystallographic data for this paper.^38*a*–*e*^ In addition, all underlying NMR and IR data supporting this work are openly available *via* the TU Graz repository at https://doi.org/10.3217/scqsy-hpx67.
