# Supplementary material for: Synthesis, structure, photochemical and electrochemical properties of α-germyl ferrocenyl ketones
Source: Dalton Trans. 2025 Nov 6;54(47):17611–20. doi: 10.1039/d5dt02029h (PMC12614158; doi:10.1039/d5dt02029h)
Supplement: DT-054-D5DT02029H-s001 [file DT-054-D5DT02029H-s001.pdf]

Supplementary Information (SI) for Dalton Transactions.
This journal is © The Royal Society of Chemistry 2025

Supporting Information

for

Synthesis, Structure, Photochemical and Electrochemical Properties of α-Germyl Ferrocenyl Ketones

Madeleine Heurix,[a] Petr Harmach,[b] Ana Torvisco,[a] Roland C. Fischer,[a] Mathias Wiech,[c] Ivana Císařová,[b] Georg Gescheidt,[c] Petr Štěpnička*[b] and Michael Haas*[a]

[a] Institute of Inorganic Chemistry, Graz University of Technology, Stremayrgasse 9/IV, 8010 Graz, Austria; [b] Department of Inorganic Chemistry, Faculty of Science, Charles University, Hlavova 2030, 128 00 Prague, Czech Republic; [c] Institute of Physical and Theoretical Chemistry, Graz University of Technology, Stremayrgasse 9/I, 8010 Graz, Austria E-mail: michael.haas@tugraz.at; stepnic@natur.cuni.cz

Table of Content

1. Copies of the NMR spectra 3
   1.1 Tris(trimethylsilyl)(ferrocenylcarbonyl)germane 3
   1.2 Tetrakis(ferrocenylcarbonyl)germane 4
   1.3 Tris(2,4,6-trimethylbenzoyl)(ferrocenylcarbonyl)germane 5
   1.4 Bis(2,4,6-trimethylbenzoyl)bis(ferrocenylcarbonyl)germane 6
   1.5 Tris(trimethylsilyl)(ferrocenylcarbonyl)silane 7
   1.6 Compound **8** 8
2. Photochemical Investigations 9
3. Photochemical Characterization of **6** 10
   3.1 Extinction Coefficients 11
   3.2 Actinometry 12
   3.3 Quantum Yields 13
4. X-ray Crystallography 14
5. Flow Reactor Setup 20
6. References 21

1. Copies of the NMR spectra

1.1 Tris(trimethylsilyl)(ferrocenylcarbonyl)germane

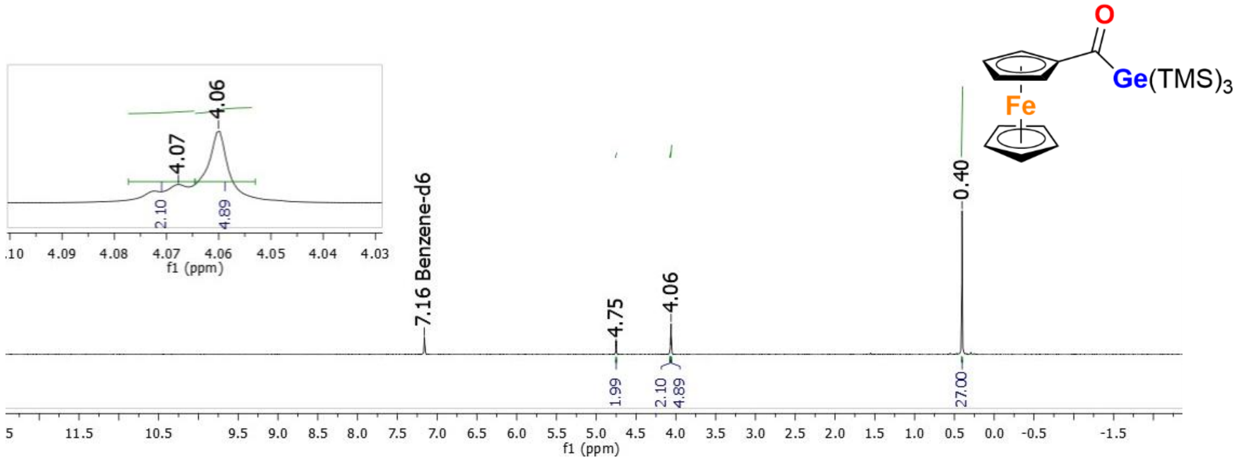

Figure S1: ^{1}H-NMR spectrum of compound **4** (C_6D_6 solution, vs ext. TMS, ppm)

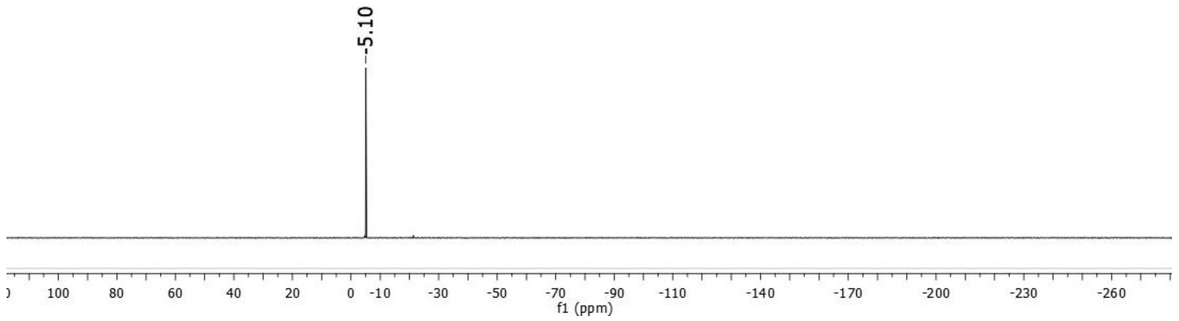

Figure S2: ^{29}Si INEPT-NMR spectrum of compound **4** (C_6D_6 solution, vs ext. TMS, ppm)

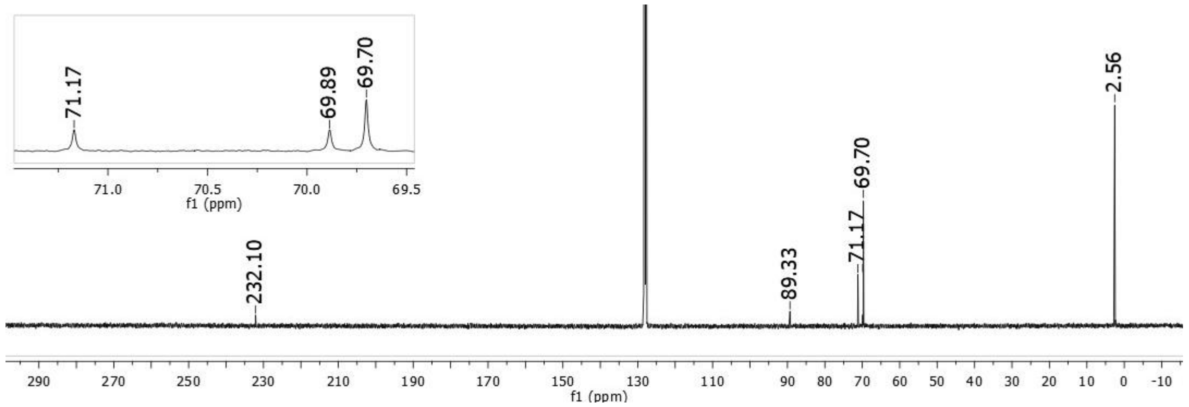

Figure S3: ^{13}C-NMR spectrum of compound **4** (C_6D_6 solution, vs ext. TMS, ppm)

1.2 Tetrakis(ferrocenylcarbonyl)germane

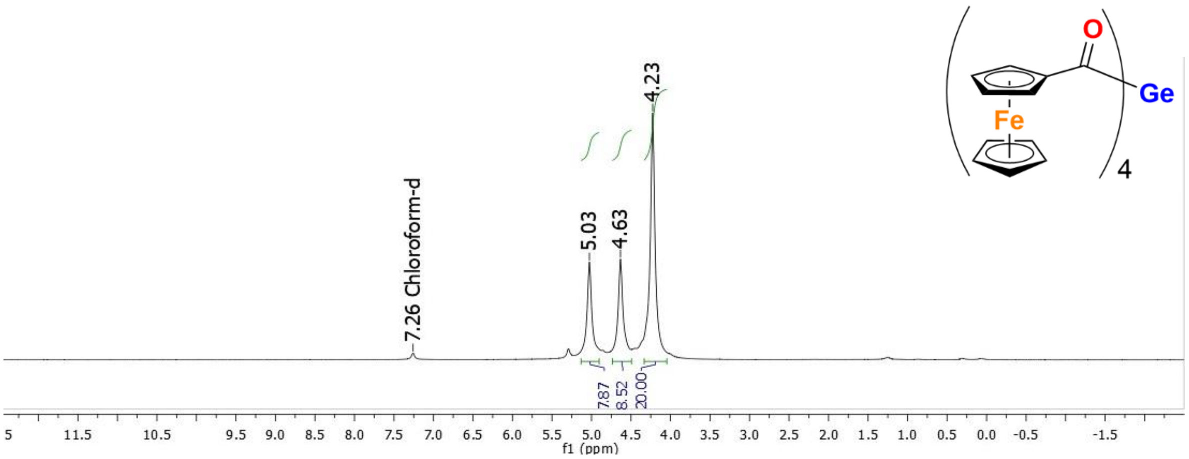

Figure S4: ^{1}H-NMR spectrum of compound **5** (C_6D_6 solution, vs ext. TMS, ppm)

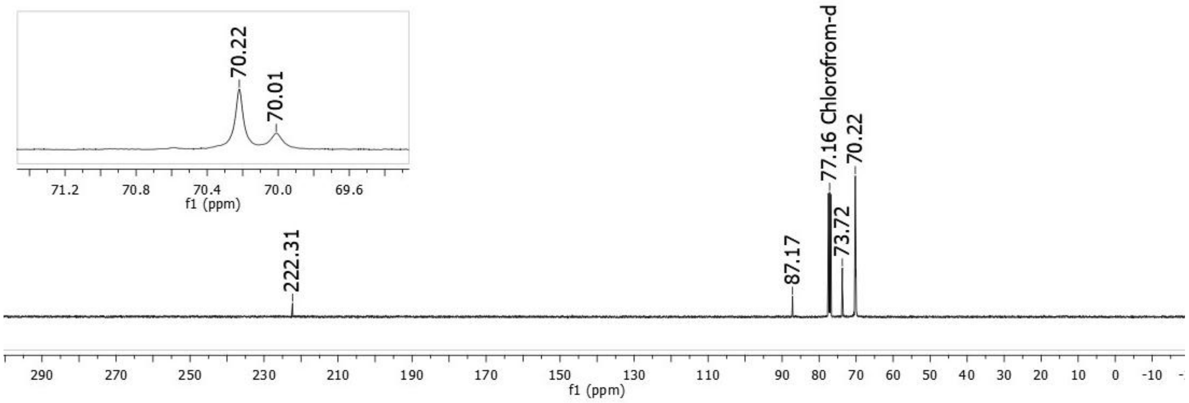

Figure S5: ^{13}C-NMR spectrum of compound **5** (C_6D_6 solution, vs ext. TMS, ppm)

1.3 Tris(2,4,6-trimethylbenzoyl)(ferrocenylcarbonyl)germane

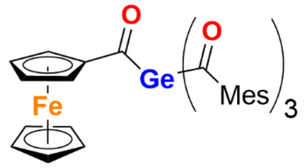

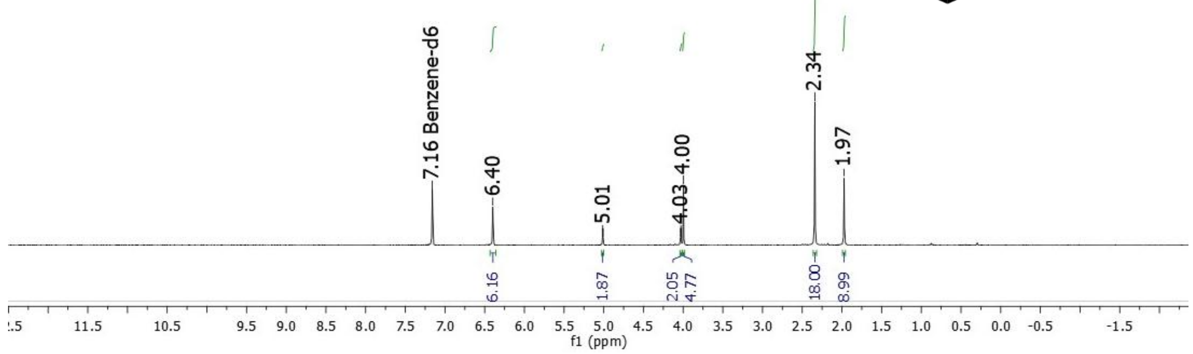

Figure S6: ^{1}H-NMR spectrum of compound **6** (C_6D_6 solution, vs ext. TMS, ppm)

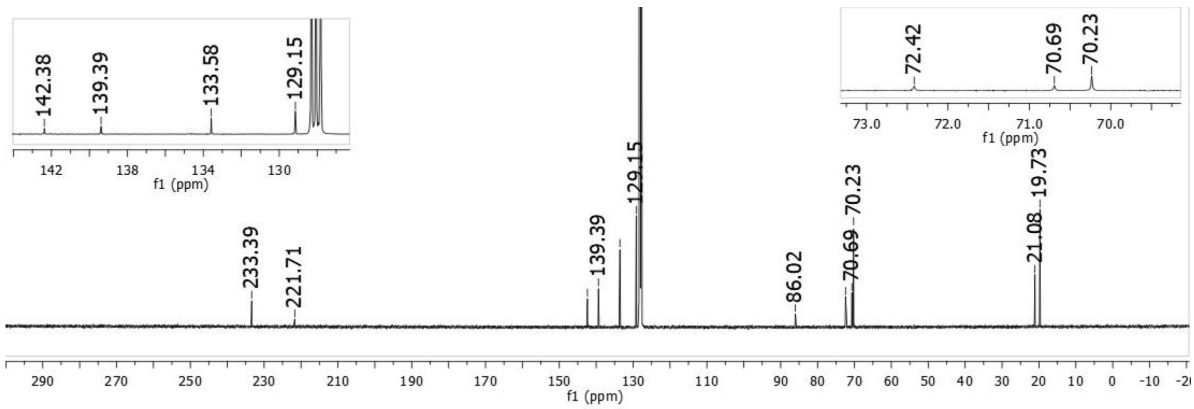

Figure S7: ^{13}C-NMR spectrum of compound **6** (C_6D_6 solution, vs ext. TMS, ppm)

1.4 Bis(2,4,6-trimethylbenzoyl)bis(ferrocenylcarbonyl)germane

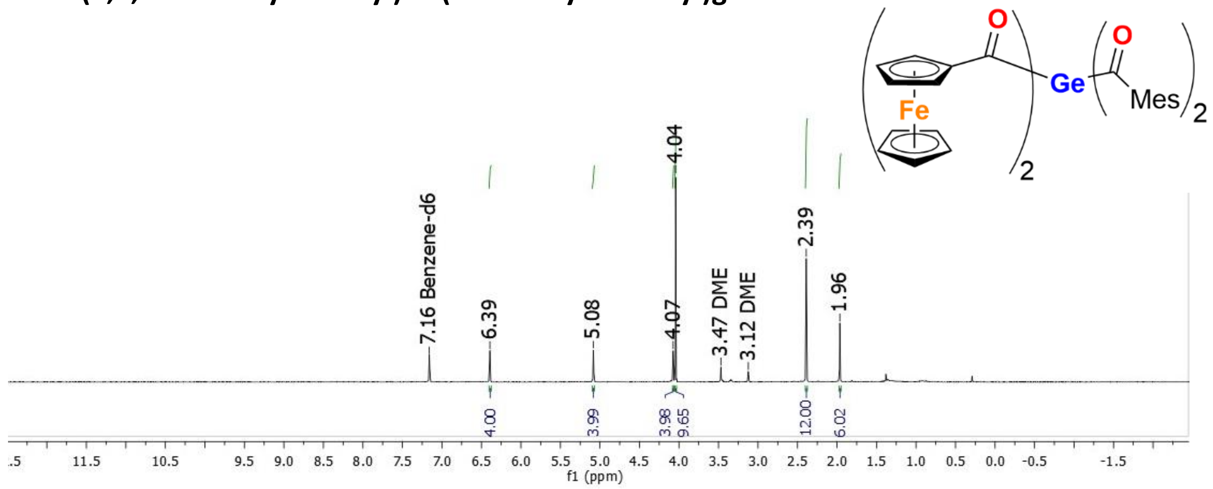

Figure S8: ^{1}H-NMR spectrum of compound **7** (C_6D_6 solution, vs ext. TMS, ppm)

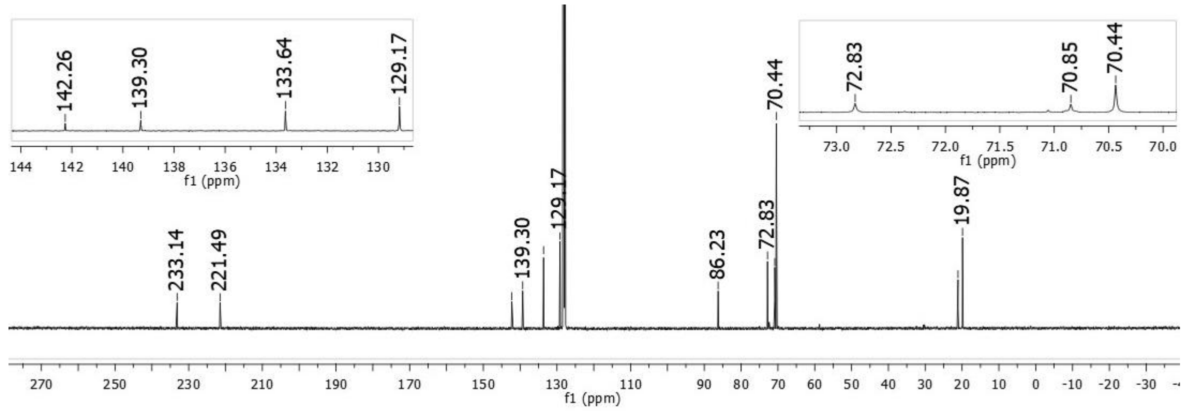

Figure S9: ^{13}C-NMR spectrum of compound **7** (C_6D_6 solution, vs ext. TMS, ppm)

1.5 Tris(trimethylsilyl)(ferrocenylcarbonyl)silane

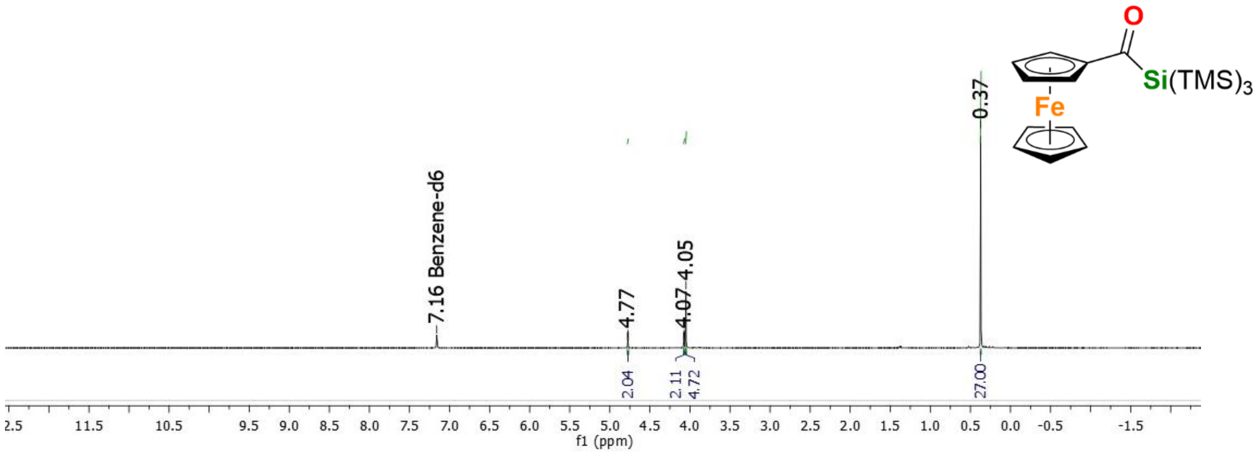

Figure S10: ^{1}H-NMR spectrum of compound **4Si** (C_6D_6 solution, vs ext. TMS, ppm)

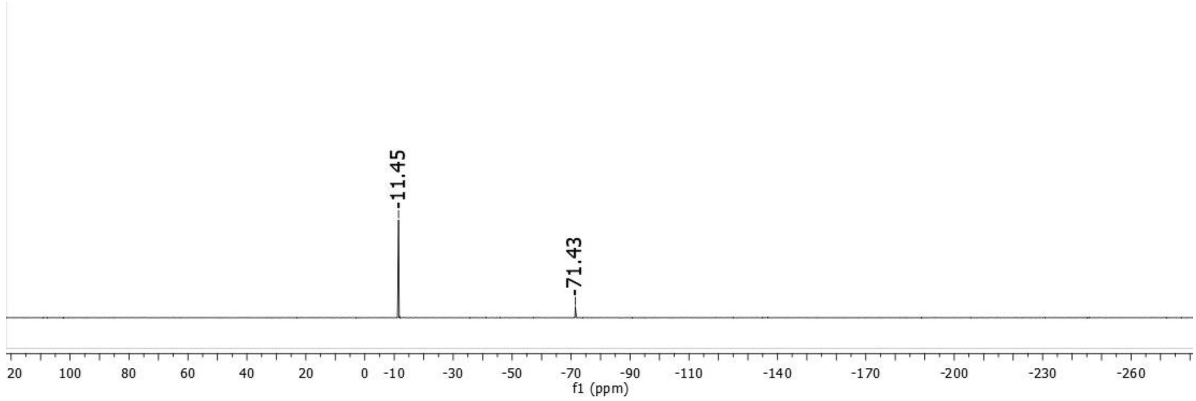

Figure S11: ^{29}Si INEPT-NMR spectrum of compound **4Si** (C_6D_6 solution, vs ext. TMS, ppm)

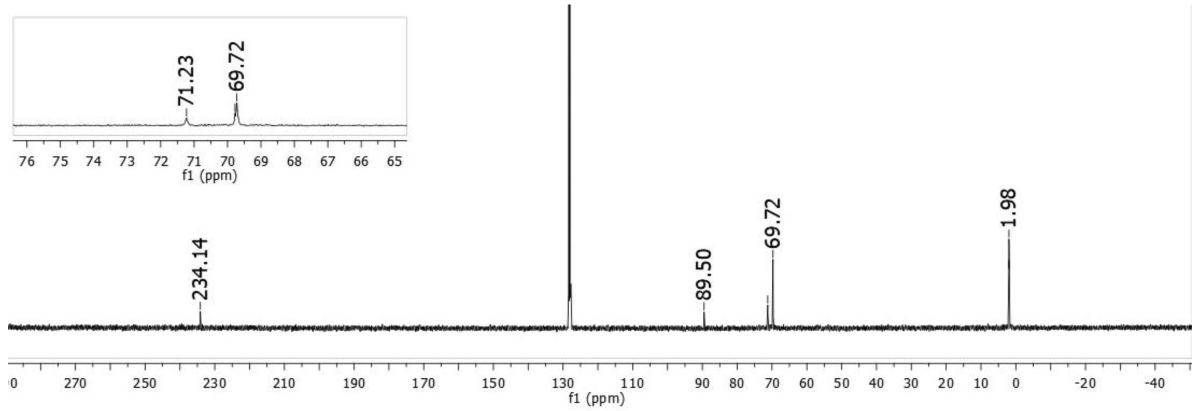

Figure S12: ^{13}C-NMR spectrum of compound **4Si** (C_6D_6 solution, vs ext. TMS, ppm)

1.6 Compound 8

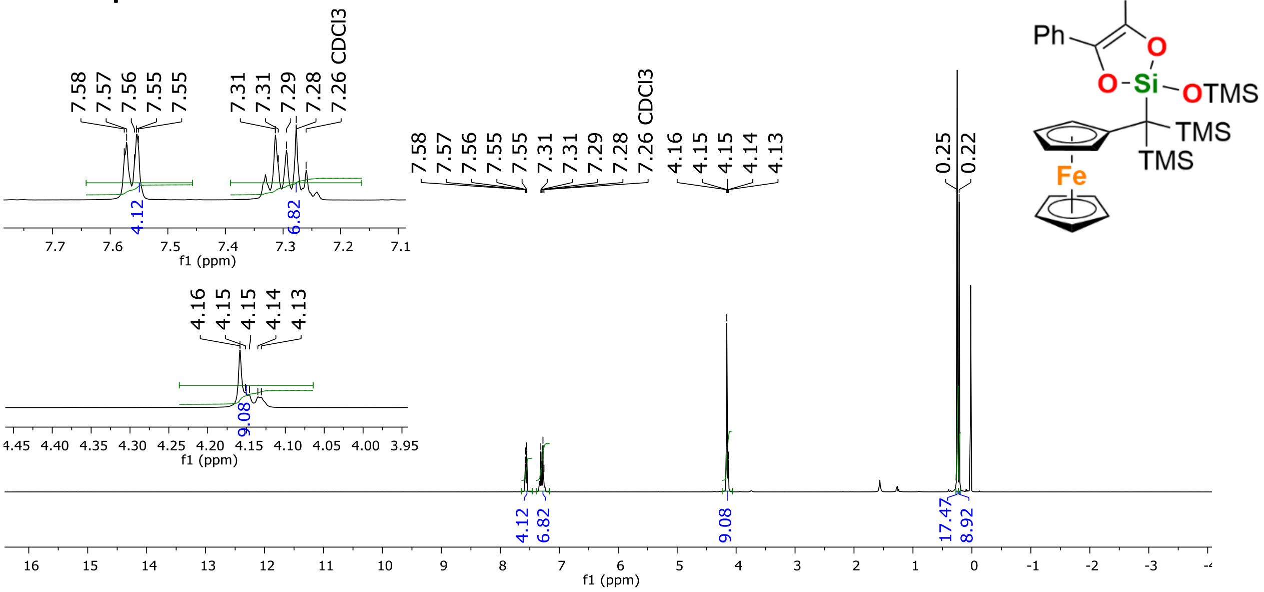

Figure 13*:* ^{1}H-NMR spectrum of compound **8** (CD$_{Cl36}$ solution, vs ext. TMS, ppm)

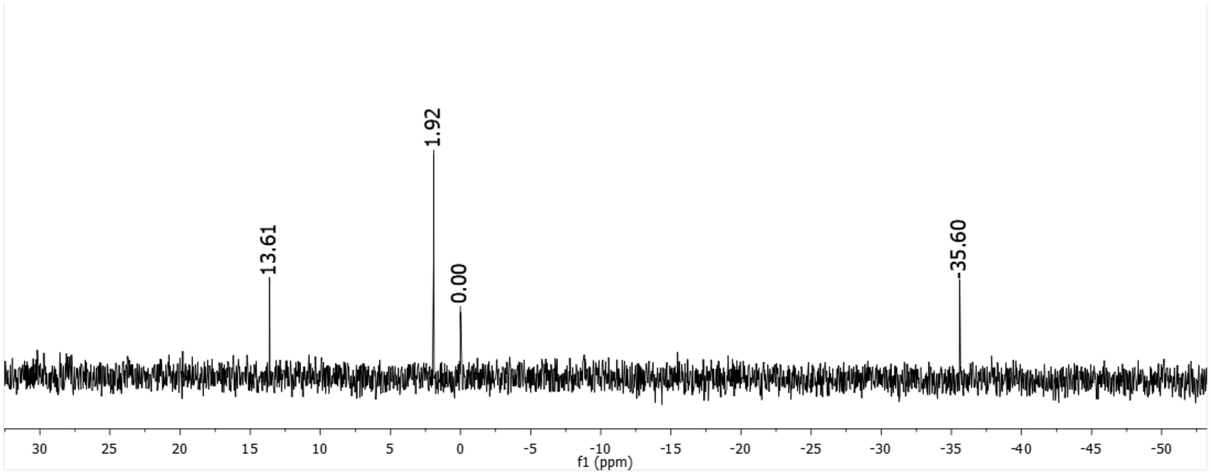

Figure 14: ^{29}Si-NMR spectrum of compound **8** ($CDCl_3$ solution, vs ext. TMS, ppm)

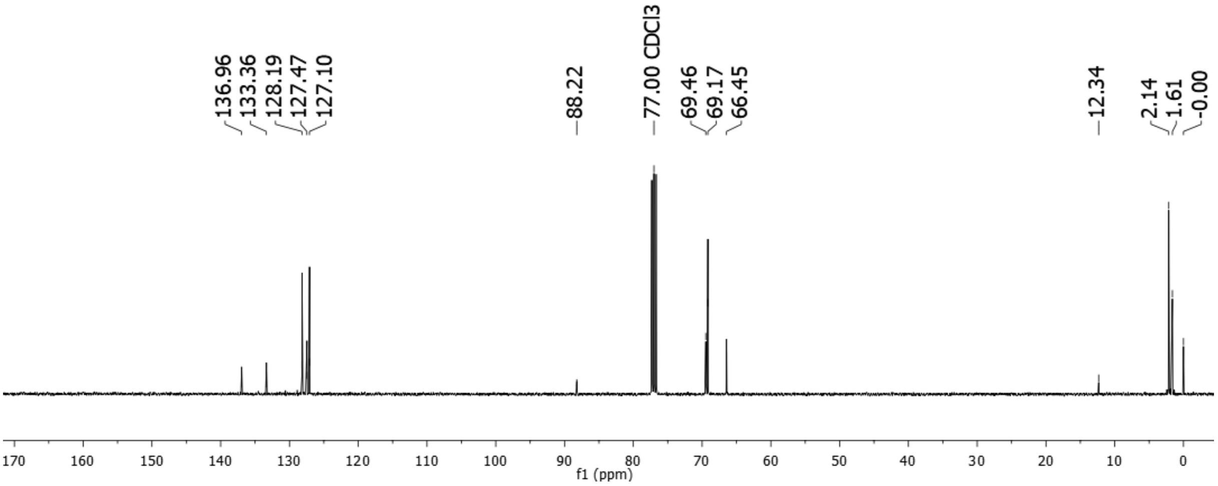

Figure 15: ^{13}C-NMR spectrum of compound **8** ($CDCl_3$ solution, vs ext. TMS, ppm)

2. Photochemical Investigations

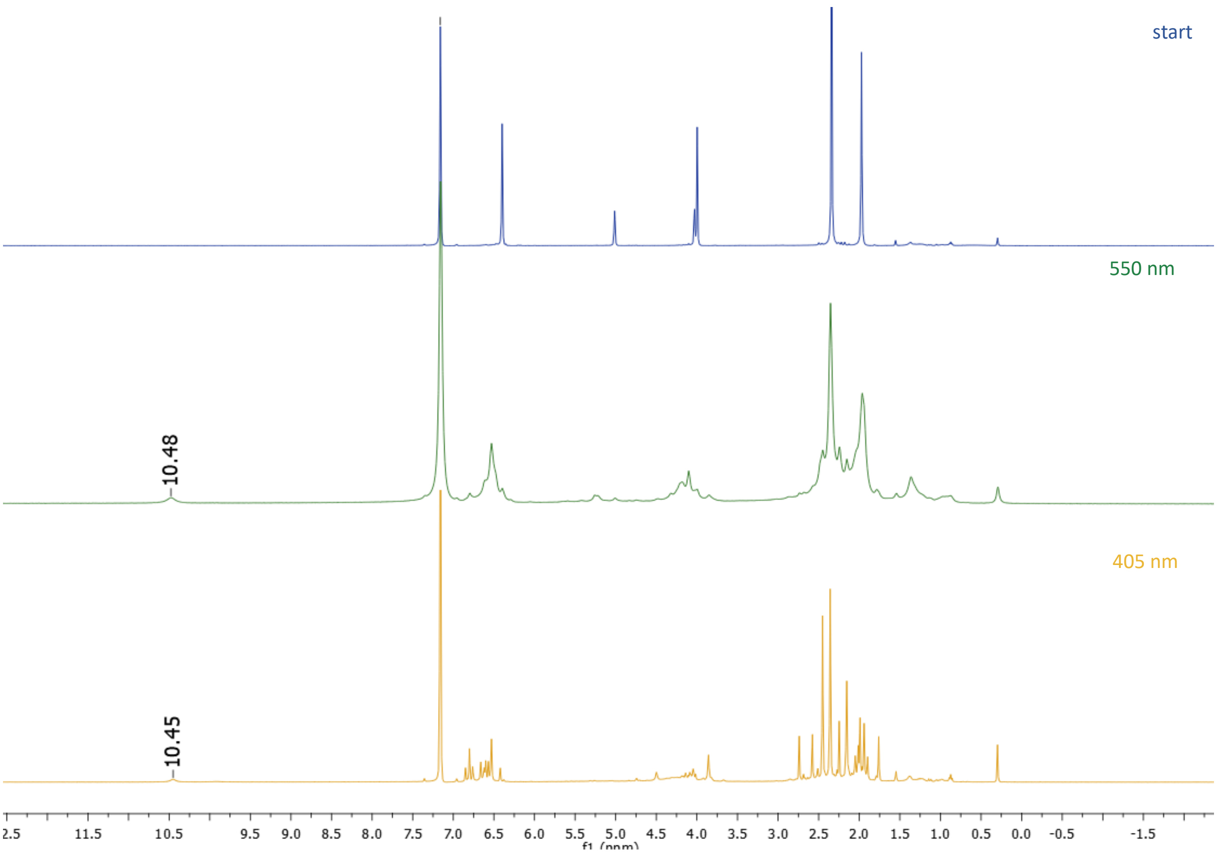

Figure S16: ^{1}H-NMR spectra of compound **6** before irradiation and after irradiation at 550 and 405 nm for 12 h

3. Photochemical Characterization of 6

The extinction coefficients and quantum yield for the light-induced α-cleavage of **6** were determined at four wavelengths between 385 nm and 500 nm. The chosen wavelengths correspond to both the n/σ → π* and π → π* transitions of the acyl and ferrocene moieties, respectively. All experiments were performed on a Tidas S500 fiber-coupled, diode array UV/Vis spectrophotometer (J&M Analytik AG, Essingen, Germany) in 10 mm fluorescence quartz cuvettes. The LEDs were characterized using a calibrated spectrophotometer (GL Spectis 1.0, GL Optic Lichtmesstechnik GmbH, Weilheim, Germany). Table S1 contains information on the emitted photon-flux, the emission maximum and the width at half height of the emission peak. Emission spectra are shown in Figure S17. A detailed description of the setup and method is given by *Stadler et al.*[1] For the 385 nm and 400 nm LEDs, the photon-flux was also determined by actinometry in accordance with the procedure reported by *Lehóczki et al.*[2]

Table S1: Optical characteristicvs of the LEDs used in the photochemical characterization of **6.**

LED	Peak Emission [nm]	Peak FWHM [nm]	Photon-Flux [mol L^{-1} s^{-1}]
1	387	15	1.15×10^{-5} [a]
2	401	16	1.18×10^{-5} [a]
3	453	22	5.56×10^{-6}
4	498	35	5.55×10^{-6}

a. value determined by chemical actinometry

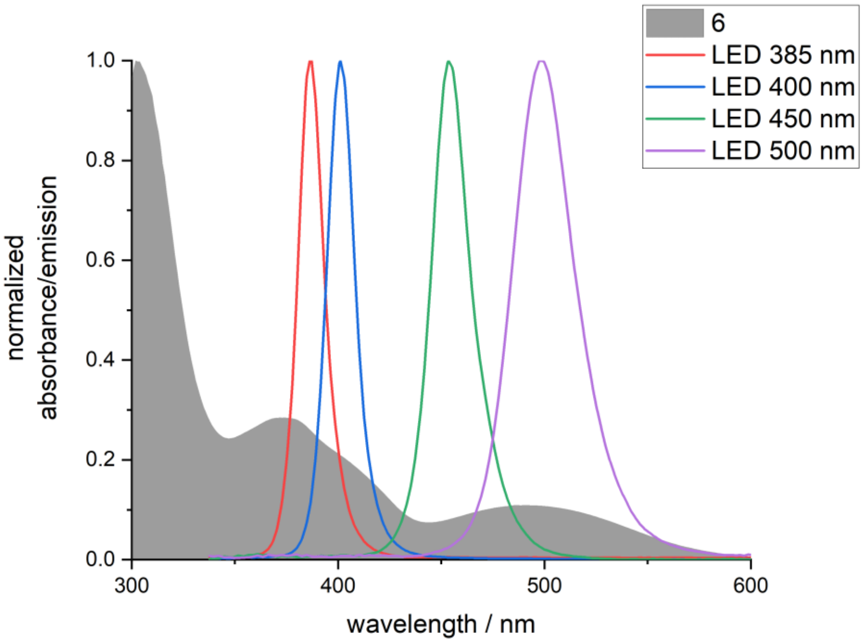

Figure S17: Emission spectra of LEDs superimposed over an absorption spectrum of **6** in THF

3.1 Extinction Coefficients

The linear dependence of the optical absorbance *A* on the extinction coefficient ε, the analyte concentration *c* and the optical pathlength *d* is given by Beer-Lambert´s law:

$$A = \varepsilon * c * d$$

Accordingly, when plotting the absorbance of sample solutions against their concentration, ε is accessible directly as the slope of a linear fit of the datapoints. We prepared sample solutions of **6** in THF in the range of 0.5 × 10^{-4} to 2.5 × 10^{-4} mol L^{-1} and plotted the experimentally determined absorbance at the wavelength of interest (corresponding to the determined LED emission maxima) against their concentration (Figure S18). The obtained extinction coefficients are given in Table S2.

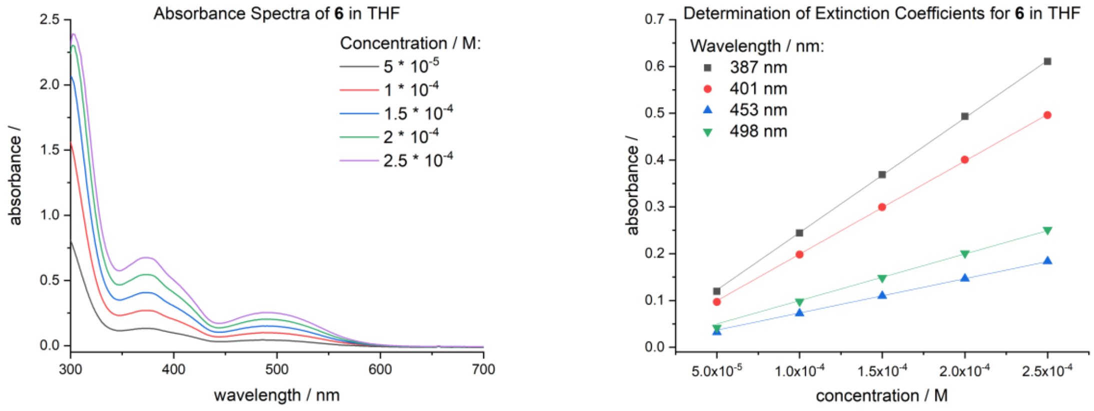

Figure S18: Left: Absorbance spectra of **6** in THF at different concentrations. Right: Absorbance plotted against sample concentration at selected wavelengths.

Table S2: Extinction coefficients ε of **6** in THF determined from the data presented above.

Wavelength [nm]	ε [L mol^{-1} cm^{-1}]
387	2.45 ± 0.01 × 10^3
401	1.99 ± 0.01 × 10^3
453	7.32 ± 0.07 × 10^2
498	9.97 ± 0.12 × 10^2

3.2 Actinometry

The photon-flux into the sample cuvettes was determined via ferrioxalate (FeOx) actinometry employing on-line UV/Vis spectroscopy. Here, 3 mL samples of 11.2 mM FeOx in 0.05 M H_2SO_4 were irradiated with LEDs using the identical setup as it is employed for the measurement of the quantum yields (see chapter 3.3). Absorbance spectra were recorded every 2 seconds for 20 minutes. The measured absorbance at 410 nm was then plotted against the irradiation time (Figure S19). The slope of a linear fit of the first 50 data points (100 s of irradiation time) yields the photon-flux I_0 via:

$$I_0 = \frac{slope}{\varepsilon_{410}\ \Phi}$$

Here, we use an extinction coefficient of ε_{410} = 146 L mol^{-1} cm^{-1} and a quantum yield of Φ = 1.2.[2] The corresponding values are given in Table S3.

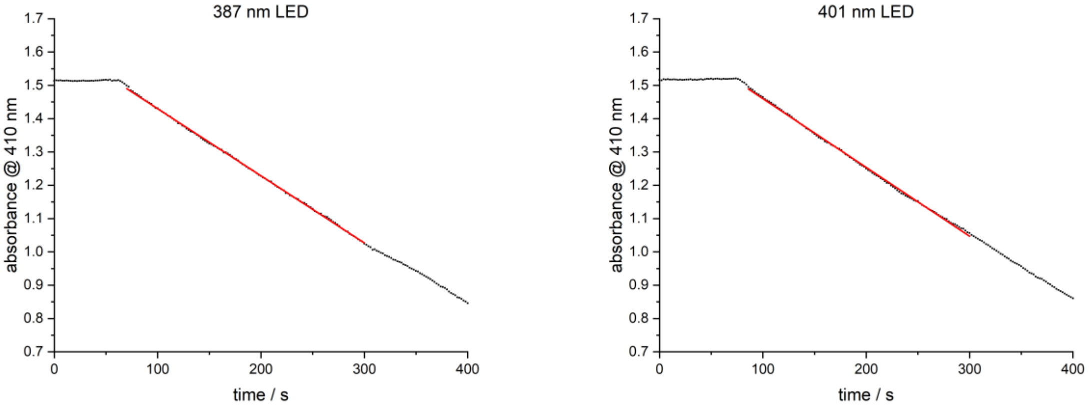

Figure S19: UV/Vis time traces of the FeOx actinometry for the 387 nm and 401 nm LEDs

Table S3: Actinometric data for calculating the photon-flux into the sample cuvette.

λ [nm]	slope [s^{-1}]	Photon-Flux [mol L^{-1} s^{-1}]
387	2.01×10^{-3}	$1.15 \pm 0.02 \times 10^{-5}$
401	2.07×10^{-3}	$1.18 \pm 0.02 \times 10^{-5}$

3.3 Quantum Yields

We used the experimentally determined extinction coefficients of **6** to prepare solutions with an absorbance of 0.05 at the irradiation wavelength. All samples were prepared in 3 mL of a 1/1 v/v mixture of THF/MMA (methyl methacrylate). Oxygen was removed from the solutions by bubbling with argon for five minutes. The solutions were irradiated with LED light perpendicular to the detection axis under stirring. Absorbance spectra were recorded every two seconds for two hours. Time traces were then obtained by plotting the absorbance at 376 nm (corresponds to the absorbance maximum of the acyl n/σ → π* transition) against the irradiation time (Figure S19). From this data, the rate constant *k* of the photobleaching was determined by a mono-exponential fit. Following the procedure outlined by Stadler *et al.*[1], the quantum yield of photobleaching Φ was obtained from:

$$\Phi = \frac{kc_0}{I_0(1-10^{-A_i})}$$

Here, c_0 is the initial concentration, I_0 is the photon-flux emitted by the LED and A_i is the initial absorbance at the irradiation wavelength. All data are summarized in Table S4.

Table S4: Data used for calculating the quantum yields for the photobleaching of **6**.

λ [nm]	c_0 [mol L^{-1}]	I_0 [$10^5 \times$ mol L^{-1} s^{-1}]	k [$10^{-3} \times$ s^{-1}]	Φ
387	2.0×10^{-5}	1.15 ± [a]	1.82 ± [a]	$3.0 \pm 0.2 \times 10^{-2}$
401	2.4×10^{-5}	1.18 ± [a]	1.22 ± [a]	$2.4 \pm 0.2 \times 10^{-2}$
453	7.8×10^{-5}	0.56	–[b]	–
498	3.6×10^{-5}	0.56	–[b]	–

a. value determined by chemical actinometry
b. bleaching rate is below the baseline drift of the instrument

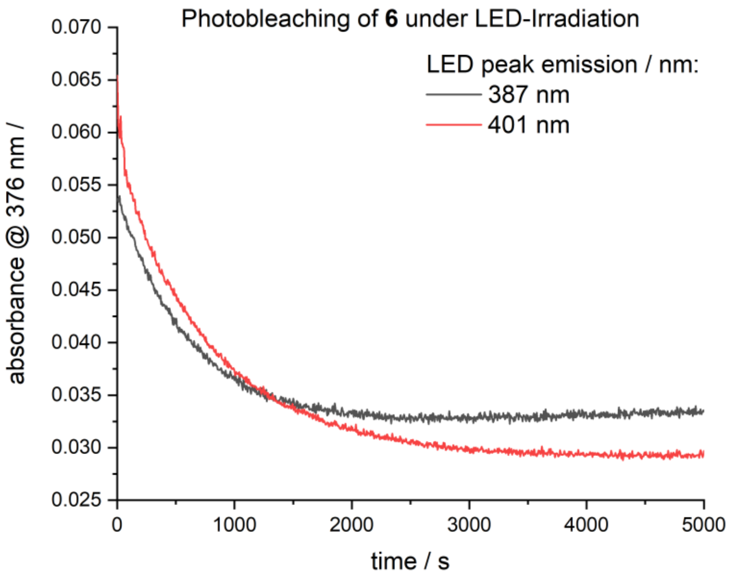

Figure S20: Photobleaching of **6** under LED-irradiation.

4. X-ray Crystallography

Crystals suitable for single crystal X-ray diffraction analysis were removed from a vial and immediately covered with a layer of silicone oil. A single crystal was selected, mounted on a copper pin, and placed in the cold N_2 stream provided by an Oxford Cryosystems Cryostream cooler. Data for the crystal structures of **4, 4Si, 5,** and **6** were collected using a Rigaku XtaLAB Synergy, Dualflex, HyPix-Arc 100 diffractometer using Cu Kα (λ = 1.54056 Å) radiation. The data for **8** were collected using a Bruker Bruker D8 VENTURE Kappa Duo diffractometer equipped with an Oxford Cryosystems Cryostream cooler; monochromated Mo Kα radiation was employed (λ = 0.71073 Å). The diffraction pattern was indexed and the total number of runs and images was based on the strategy calculation from the program CrysAlisPro.[3] The unit cell was refined and data reduction, scaling and absorption corrections were performed using CrysAlisPro. Using Olex2.[4] The structures were solved with the SHELXT[5] structure solution program and refined using the SHELXL[6] refinement package and full-matrix least squares minimization based on F^2. All non-hydrogen atoms were refined anisotropically. Hydrogen atom positions were calculated geometrically and refined using the riding model. Disorder was handled by modelling the occupancies of the individual orientations using free variables to refine the respective occupancy of the affected fragments (PART).[7] In some cases, the similarity SAME restraint, the similar-ADP restraint SIMU, and the rigid-bond restraint DELU and RIGU, as well as the constraints EXYZ and EADP were used in modelling disorder to make the ADP values of the disordered atoms more reasonable. In some cases, the distances between arbitrary atom pairs were restrained to possess the same value using the SADI instruction, and certain target values were used. In some tough cases of disorder, anisotropic displacement factors were restrained (ISOR) to behave more isotropically. In **6**, a disordered Cp ring and a C=O moiety were refined using split positions, 80/20 and 70/30, respectively. In **5**, a solvent mask was calculated[1] to remove the disordered solvent of crystallization, CH_2Cl_2. The crystals of **8** suffered from defects reflected as residual electron density corresponding to a partial disorder of molecule 2 (Fe2) from which only the heavy atoms (Si, Fe) could be identified for the less populated orientation (approximately 6% occupancy) due to overlaps. Therefore, the minor orientation, resulting in several notable residual electron density peaks, could not be unambiguously included in the refinement.

All crystal structure representations were made with the program Diamond.[8] CIF files were edited, validated, and formatted either with the programs encifer,[9] publCIF,[10] or Olex2. CCDC 2478329 (**4**), 2478328 (**4Si**), 2478330 (**5**), 2478331 (**6**), and 2478332 (**8**) contain the supplementary crystallographic data. These data can be obtained free of charge from The Cambridge Crystallographic Data Centre *via* www.ccdc.cam.ac.uk/data_request/cif. Table S5 contains relevant crystallographic data and details of the measurement and structure refinement.

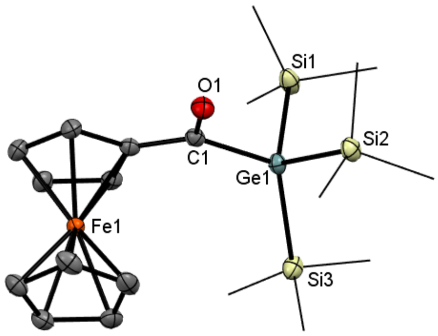

Figure S21. ORTEP representation of compound **4.** Thermal ellipsoids are depicted at the 50 % probability level. Hydrogen atoms are omitted for clarity. Selected bond length (Å) and bond angles (deg) with estimated standard deviations: Ge(1)-C(1) 2.3913(3), Ge(1)-Si(1) 2.3861(4), Ge(1)-Si(2) 2.3913(3), Ge(1)-Si(3) 2.3877(4), C(1)-O(1) 1.2256(12), C(1)-C(2) 1.4703(16).

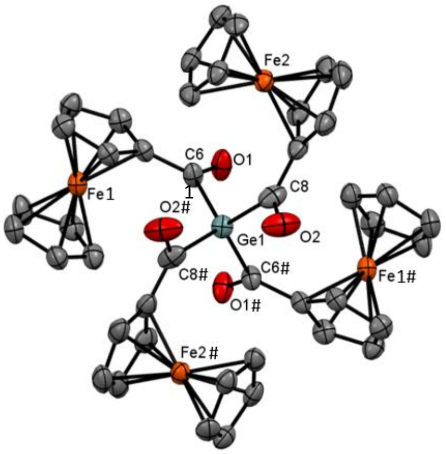

Figure S22. ORTEP representation of compound **5.** Thermal ellipsoids are depicted at the 50 % probability level. Hydrogen atoms are omitted for clarity. Selected bond length (Å) and bond angles (deg) with estimated standard deviations: Ge(1)-C(6#) 2.025(4), Ge(1)-C(8#) 2.018(5), O(1#)-C(6#) 1.221(5), O(2#)-C(8#) 1.232(7), C(6#)-C(16#) 1.454(6), C(8#)-C(14#) 1.450(8).

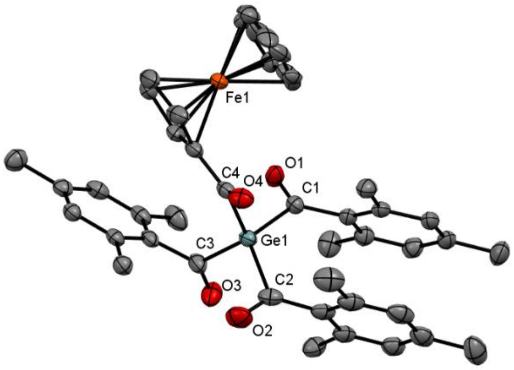

Figure S23. ORTEP representation of compound **6.** Thermal ellipsoids are depicted at the 50 % probability level. Hydrogen atoms are omitted for clarity. Selected bond length (Å) and bond angles (deg) with estimated standard deviations: Ge(1)-C(1) 2.053(16), Ge(1)-C(2) 2.085(18), Ge(1)-C(3) 2.0451(17), Ge(1)-C(4) 2.0278(16), O(1)-C(1) 1.2147 (19); O(2)-C(2) 1.2110(2), O(3)-C(3) 1.221(6), O(4)-C(4) 1.223(2), C(1)-C(5) 1.497(2), C(2)-C(14) 1.499(3), C(3)-C(23) 1.490(2), C(4)-C(32) 1.458(2).

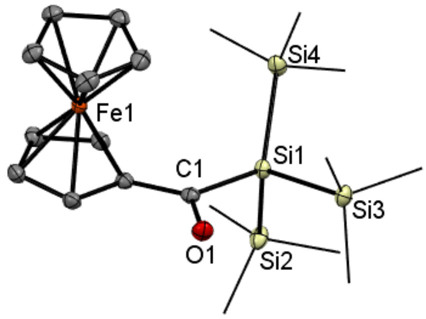

Figure S24: ORTEP representation of compound **4Si**. Thermal ellipsoids are depicted at the 50 % probability level. Hydrogen atoms are omitted for clarity. Selected bond lengths (Å) and bond angles (deg) with estimated standard deviations: Si(1)-C(1) 1.9479(12), Si(1)-Si(2) 2.3546(5), Si(1)-Si(3) 2.3582(4), Si(1)-Si(4) 2.3578(4), C(1)-O(1) 1.2337(14), C(1)-C(7) 1.4723(16)

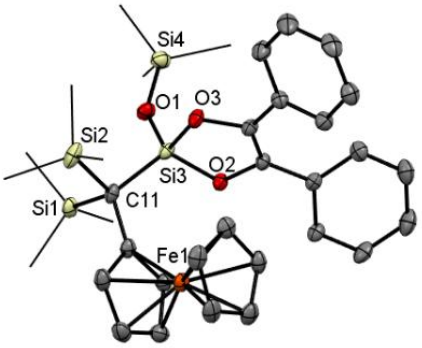

Figure S25: ORTEP representation of compound **8**, molecule 1. Thermal ellipsoids are depicted at the 50 % probability level. Hydrogen atoms are omitted for clarity. Selected bond lengths (Å) and bond angles (deg) with estimated standard deviations: Si(3)-O(1) 1.6053(19), Si(3)-O(2) 1.6659(18), Si(3)-O(3) 1.6668(19), Si(3)-C(11) 1.844(3), Si(1)-C(11) 1.9360(3), Si(2)-C(11) 1.9190(3), Si(4)-O(1) 1.6500(2).

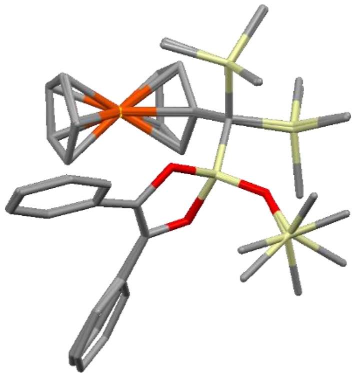

Figure S26: Least-squares overlap of the two crystallographically independent molecules in the crystal structure of **8**.

Table S5: Crystallographic data and details measured for **4, 5 6**, **8** and **4Si**

**Compound**	**4**	**5**	**6**	**8**	**4Si**
Formula	$C_{20}H_{36}FeGeOSi_3$	$C_{44}H_{36}Fe_4GeO_4$	$C_{34}H_{46}FeO_3Si_4$	$C_{41}H_{42}FeGeO_4$	$C_{20}H_{36}FeOSi_4$
FW (g mol^{-1})	505.20	924.72	670.92	727.18	460.12
T (K)	100(2)	100(2)	100(2)	100(2)	100(2’)
Crystal system	Tetragonal	Monoclinic	triclinic	Orthorhombic	tetragonal
Space group	$I4_1/a$	$C2/c$	P–1	$Pbca$	$I4_1/a$
a (Å)	18.27024(4)	14.0890(3)	11.0624(7)	18.39641(10)	18.24740(10)
b (Å)	18.27024(4)	16.1723(2)	17.8802(13)	14.34083(10)	18.24740(10)
c (Å)	30.63623(11)	21.2268(4)	18.5402(13)	26.21799(18)	30.3442(3)
α (°)	90	90	91.662(3)	90	90
β (°)	90	101.9230(19)	105.985(3)	90	90
γ (°)	90	90	91.460(3)	90	90
V (Å^3)	10226.42(6)	4732.21(15)	3521.8(4)	6916.82(8)	10133.6
Z	16	4	4	8	16
Crystal size (mm)	0.27 × 0.24 × 0.17	0.19 × 0.14 × 0.11	0.19 × 0.19 × 0.16	0.20 × 0.10 × 0.09	0.20 x 0.17 x 0.13
Crystal habit	Block, orange	Block, red	Prism, orange	Block, red	Block, orange
d_{calc} (Mg m^{-3})	1.313	1.298	1.265	1.397	1.208
μ (mm^{-1})	7.389	10.60	0.596	4.76	6.632
Radiation type	Cu K_α	Cu K_α	Mo K_α	Cu K_α	Cu K_α
Wavelength (Å)	1.54056	1.54056	0.71073	1.54056	1.54056
ϑ range (°)	2.8–80.0	4.2–74.3	2.2-28.3	3.4–79.2	2.8-77.8
F(000)	4224	1872	1424	3024	3936
T_{min}, T_{max}	0.199, 1.000	0.589, 0.811	0.895, 0.911	0.727, 1.000	0.347, 1.000

R_{int}	0.033	0.062	0.033	0.038	0.022
No. of independent and observed [$I > 2\sigma(I)$] reflections	5221, 5183	5010, 4168	17463, 15836	6219, 5791	43158, 5352, 5074
No. of parameters, restraints	245, 0	240, 0	775, 0	489, 450	245,0
$\Delta\rho_{max,min}$ (e Å^{-3})	0.27, –0.17	0.79, –0.65	2.75, –1.02	0.28, –0.25	0.28, -0.21
GooF	1.13	1.07	1.06	1.03	1.035
$R1$, $wR2$ (all data)	0.0171, 0.0423	0.0686, 0.1677	0.0630, 0.1521	0.0267, 0.0611	0.0244, 0.0548
$R1$, $wR2$ (observed data)	0.0169, 0.0422	0.0577, 0.1598	0.568, 0.1458	0.0240, 0.0598	0.0214, 0.0540

5. Flow Reactor Setup

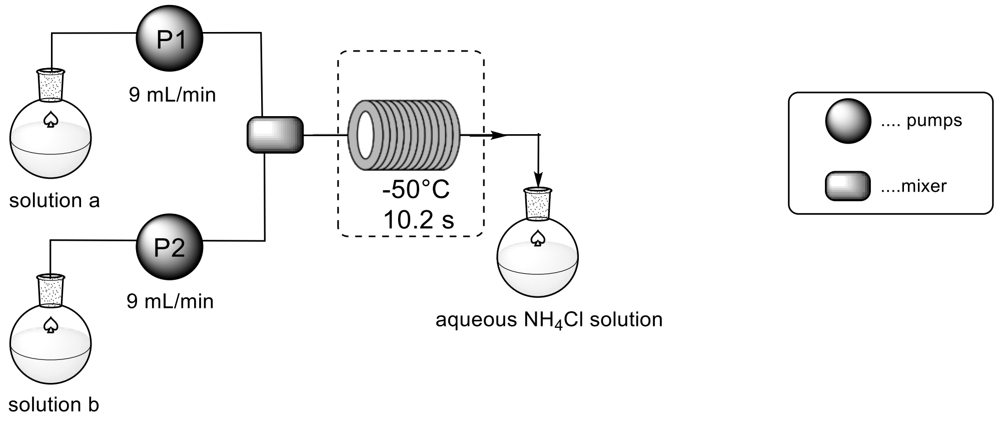

Figure S27: Setup for the continuous flow reactor

A Syrris Asia reciprocating syringe pump was used to deliver both component streams (solution 1 and solution 2). The pump was equipped with syringes of 5 mL and 2.5 mL volume (0.05-10 mL min^{-1} flow rate; wetted parts: PTFE and glass). Connections between the pumps and mixer were made of 1/16" outer diameter (0.8 mm inner diameter) PFA tubing, connected with 1/4" PTFE finger thight fittings. Reactors of 2-6 mL volume were made using 1/16" outer diameter PFA tubing (0.8 mm inner diameter). To mix the two solutions, a Y-mixer (PEEK), with a 0.5 mm aperture, was used. For the synthesis of **2,** a total flow rate of 18 mL min^{-1} and a reactor volume of 3.07 mL were used.

6. References

1. Stadler E.; Eibel A.; Fast D.; Freißmuth H.; Holly C.; Wiech M.; Moszner N.; Gescheidt G.; *Official journal of the European Photochemistry Association and the European Society of Photobiology* **2018**, *17* (5), 660-669. doi: 10.1039/c7pp00401j

2. Lehóczki T.; Józsa É.; Ősz K,; *Journal of Photochemistry and Photobiology A: Chemistry* **2013**, *251,* 63-68. doi: 10.1016/j.jphotochem.2012.10.005

3. *CrysAlisPro Software System*, 1.171.43.92a; Rigaku Oxford Diffraction: 2023.

4. Dolomanov, O. V.; Bourhis, L. J.; Gildea, R. J.; Howard, J. A. K.; Puschmann, H., OLEX2: a complete structure solution, refinement and analysis program. *J. Appl. Crystallogr.* **2009,** *42* (2), 339-341. doi:10.1107/S0021889808042726

5. Sheldrick, G. M., SHELXT - Integrated space-group and crystal-structure determination. *Acta Crystallogr., Sect. A: Found. Adv.* **2015,** *71* (1), 3-8. doi:10.1107/S2053273314026370

6. Sheldrick, G. M., A short history of SHELX. *Acta Crystallogr., Sect. A: Found. Adv.* **2008,** *64* (1), 112-122. 10.1107/S0108767307043930

7. Müller, P.; Herbst-Irmer, R.; Spek, A. L.; Schneider, T. R.; Sawaya, M. R., *Crystal Structure Refinement: A Crystallographer's Guide to SHELXL* Oxford University Press: 2006; p 232.

8. Putz, H.; Brandenburg, K. *Diamond - Crystal and Molecular Structure Visualization*, 4.6.5; Crystal Impact: Bonn, Germany.

9. Allen, F. H.; Johnson, O.; Shields, G. P.; Smith, B. R.; Towler, M., CIF applications. XV. enCIFer: a program for viewing, editing and visualizing CIFs. *J. Appl. Crystallogr.* **2004,** *37* (2), 335-338. doi:10.1107/S0021889804003528

10. Westrip, S., publCIF: software for editing, validating and formatting crystallographic information files. *J. Appl. Crystallogr.* 2010, *43* (4), 920-925. doi:10.1107/S0021889810022120
